# Natural xanthones as α-Mangostin induce vasorelaxation involving key gating residues in the S6 domain of BK channels

**DOI:** 10.7554/eLife.109479

**Published:** 2026-05-06

**Authors:** Soenke Cordeiro, Robert Patejdl, Thomas Baukrowitz, Marianne A Musinszki

**Affiliations:** 1 Institute of Physiology, Christian-Albrechts-University Kiel Kiel Germany; 2 Department of Human Medicine, Health and Medical University Erfurt Erfurt Germany; Universidad Nacional Autónoma de México Mexico; Stanford University United States

**Keywords:** BK, mangostin, vasorelaxation, VSMC, Slo1, KCa1.1, Mouse

## Abstract

Polyphenolic compounds are widely explored for health benefits, including hypertension, but their active ingredients, molecular targets, and mechanisms remain poorly defined. We identify the xanthone Mangostin from *Garcinia mangostana* as a potent modulator of several potassium channels, with large-conductance K^+^ (BK) channels as its primary target for vasorelaxation. Mangostin-activated BK channels as α subunits alone, in complexes with vascular β1 subunits, and in reconstituted BKα/β1–Ca_v_ nanodomains. It shifted BK voltage activation to more negative potentials by antagonizing channel closure and promoting channel opening without markedly altering Ca²^+^ sensitivity. Docking, competition, single-channel analysis, and mutagenesis localized the binding site in the pore cavity below the SF, involving gating-critical S6 residues I308, L312, and A316, and suggest that Mangostin stays bound in closed and open states. These findings establish BK channel activation as the core molecular mechanism driving Mangostin’s vascular effects and define its structural mode of action, informing nutraceutical safety assessment and BK-targeted drug design.

## Introduction

Nutraceuticals (superfoods, dietary supplements, and traditional remedies) promise a plethora of health benefits that are usually not definitely proven by clinical studies. Well-known examples are resveratrol present in grapes and red wine (Jang et al., 1997; Brown et al., 2024), carotenes and tocopherols (Alpha-Tocopherol and Beta Carotene Cancer Prevention Study Group, 1994; Hennekens et al., 1996; Xin et al., 2022), or the recent resurgence of quercetin (Lee et al., 2021; Di Pierro et al., 2022). In evaluating clinical relevance and exploring pharmacological opportunities of such phytochemicals, obstacles are often the identification of the biologically active substance in complex matrices of plant secondary metabolites and the lack of a definite cellular determinant that could link a biologically active substance to a physiological process. However, a clear molecular target and mechanism of action that can explain its effects is the prerequisite to further drug development.

Natural polyphenols are known to modulate diverse potassium (K^+^) channel families. For example, various members of the flavonoid subgroup activate K_Ca_ (BK) channels as well as several K_v_, K_ir_, and K_2P_ channels, including TREK-1 channels (Gierten et al., 2008; Nardi and Olesen, 2008; Kim et al., 2011; for a comprehensive review see Richter-Laskowska et al., 2023). A recent study suggested that α-Mangostin, a xanthone from *Garcinia mangostana,* modulates ion channels and binds in the pore cavity of TREK K_2P_ channels (Kim et al., 2023). α- and γ-Mangostins are the main xanthones found in the mangosteen fruit pericarp, whose ethanolic extracts are used as traditional medicine and consumed as nutraceuticals. They are thought to exert a plethora of health-promoting effects, such as cardioprotective (Eisvand et al., 2022), analgesic (Kim et al., 2023), antioxidant (Kong et al., 2022), anti-inflammatory (Kim, 2021), antidiabetic (John et al., 2022), antifibrotic (Li et al., 2019), antimicrobial (Tatiya-Aphiradee et al., 2016), and antiproliferative effects (Majdalawieh et al., 2024). Interestingly, very recent research reported that α-Mangostin has antihypertensive properties, and it decreased systolic and diastolic blood pressure in a rat model (Xu et al., 2024). Earlier studies had demonstrated the relaxation of aortic rings upon Mangostin incubation, but remained somewhat contradictory as to the site of action and the molecular target (Chairungsrilerd et al., 1996; Chairungsrilerd et al., 1997; Tep-Areenan and Suksamrarn, 2012). Hence, the molecular mechanism of this antihypertensive effect of Mangostin remains unclear.

Major potassium channels present in vascular smooth muscle are Ca^2+^-activated BK channels, also known as MaxiK, Slo1, or K_Ca_1.1 channels (Wu and Marx, 2010; Tykocki et al., 2017; Pereira da Silva et al., 2022). They are synergistically activated by intracellular calcium (Ca_i_^2+^) and voltage, and they play a crucial role in the regulation of vascular smooth muscle tone, with implications for blood pressure regulation. BK channels act as feedback regulators, which counteract depolarization and balance the elevation of Ca_i_^2+^ through voltage-dependent L-type Ca^2+^ channels. The pore-forming BKα subunit is expressed ubiquitously, and tissue-specific properties are conveyed by regulatory β-, γ-, and LINGO subunits (Latorre et al., 2017; Gonzalez-Perez and Lingle, 2019; Dudem et al., 2020). In vascular smooth muscle cells, BKα assembles with the β1 subunit, which enhances its apparent Ca_i_^2+^ sensitivity, decreases voltage sensitivity (Dworetzky et al., 1996; Brenner et al., 2000; Orio and Latorre, 2005; Li and Yan, 2016), and modulates its pharmacological properties (McManus et al., 1995; Hanner et al., 1997).

Structurally, BK channels are homotetramers consisting of the pore-forming α subunits with seven transmembrane segments (S0–S6), a linker region, and two intracellular RCK domains. The RCK domains and their interface to the transmembrane segments contain different Ca_i_^2+^ binding sites, S1–S4 comprise the voltage-sensing domain, and S5 and S6 together with the pore helix constitute the pore domain. Both depolarization or Ca_i_^2+^ elevation can open the pore via allosteric mechanisms, and this regulation, as well as the modulation by mutations, small molecules, and regulatory subunits, can be described well by an allosteric gating model (Horrigan and Aldrich, 2002; Latorre et al., 2017). However, the structural determinants that underlie this allosteric modulation remain unclear. The S6 segment has been shown to undergo conformational changes during activation, and several gating-sensitive residues, as well as modulator binding sites for molecules of surprisingly different chemical structure, are known in this pore region or the adjacent linker to the cytoplasmic C-terminus (Nardi and Olesen, 2008; Zhou et al., 2011; Roy et al., 2012; Hoshi et al., 2013b; Chen et al., 2014; Webb et al., 2015; Hoshi and Heinemann, 2016; Schewe et al., 2019; Rockman et al., 2020; Gonzalez-Sanabria et al., 2025). Investigations of the activation mechanism indicated that these substances act without directly affecting the voltage- or Ca^2+^ sensory domains, probably by modifying the close interactions of S5 and S6 with the voltage sensor bundle and the gating ring that enable opening of the channel.

Small molecule BK channel activators are promising to treat diverse diseases like fragile X syndrome, overactive bladder, pulmonary hypertension, chronic obstruction, erectile dysfunction, and reperfusion injury; however, no prospective drug successfully completed a phase III trial yet (Bentzen et al., 2014; Barenco-Marins et al., 2025; Ferraguto et al., 2024). Therefore, adding a pharmacophore derived from the xanthone group may aid in developing future drug candidates.

We used a combination of functional studies in cultured cells and vascular tissue, as well as molecular docking, to show that BK channels are a molecular target of Mangostins and that they mediate vascular relaxation observed upon Mangostin application. BK channels were opened most prominently among the K^+^ channel representatives tested and were activated by α-Mangostin as well as by an extract of the mangosteen pericarp marketed as a nutraceutical. Investigation of the activation mechanism showed that α-Mangostin essentially facilitates BK channel activation by shifting its voltage dependence, modulating gating kinetics, and enhancing the open probability, without changing the Ca_i_^2+^ sensitivity. These effects were due to direct binding to the pore-forming BKα subunit. Competition experiments, molecular docking, and scanning mutagenesis identified the binding region in S6 just below the selectivity filter (SF). Mangostin activation was preserved in reconstructed nanodomains with Ca_v_ channels, which cause local elevation of intracellular Ca_i_^2+^, mimicking the physiological situation in smooth muscle cells. Finally, we show that BK channels specifically mediate relaxation of aortic preparations from mice. Our study explains the molecular activation of BK channels by a natural xanthone and sheds light on the mechanism underlying one of its claimed health benefits.

## Results

### α-Mangostin particularly activates BKα and BKα/β1 channels

**Figure 1.**
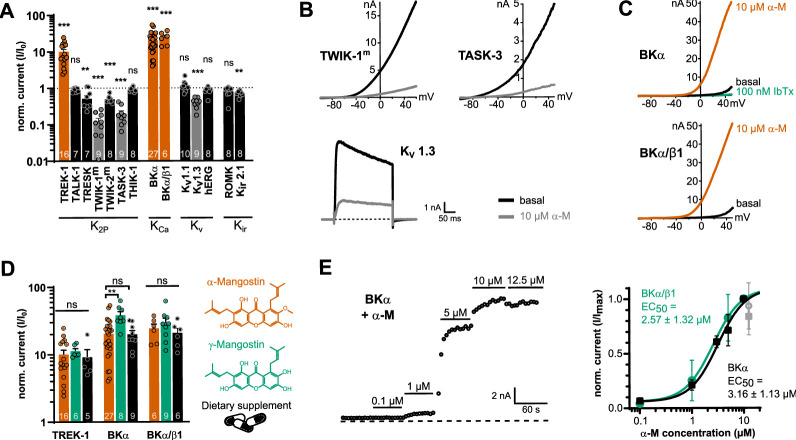
Mangostin potently activates BKα and BKα/β1 channels compared to other potassium channel representatives. (**A**) Current fold change ± SEM of currents of representatives from different potassium channel families upon application of 10 µM α-Mangostin. K_2P_ and K_Ca_ channel currents were recorded with ramp protocols from –100 to +50 mV and analyzed at +40 mV; K_v_1.1 and K_v_1.3 channel currents were evoked using a rectangle pulse to +40 mV, and hERG currents were recorded with a rectangle pulse to +60 mV followed by hyperpolarization to –120 mV, which was analyzed. K_ir_ channels were recorded using ramp protocols from –150 to +50 mV, and currents were analyzed at –140 mV. All measurements were made in transiently transfected HEK293 cells in physiological potassium gradients with 100 nM intracellular free Ca^2+^ with a holding potential of –80 mV. TWIK-1^m^ and TWIK-2^m^ denote channels where the retrieval motif was removed to improve membrane expression, and intracellular K^+^ was exchanged for Rb^+^ to enhance currents. (**B**) Representative current traces of channels that were inhibited more than 60% by 10 µM α-Mangostin. (**C**) Representative current traces of BKα and BKα/β1 channels activated by 10 µM α-Mangostin (α-M). (**D**) Current fold change ± SEM after application of α-Mangostin, γ-Mangostin, and a dietary supplement to TREK-1, BKα, and BKα/β1 channels. Currents were recorded and analyzed as in (**A**). (**E**) Representative time course of the dose-dependent activation of BKα channels by increasing concentrations of α-Mangostin (left) and the resulting dose–response relationships for BKα and BKα/β1 channels (right). Currents were recorded and analyzed as in (**A**); the grey data point at 12.5 µM was not included in the Hill fit. Data and statistics see Source data file 1.

In previous studies, α-Mangostin was shown to activate members of the K_2P_ channel family, while other K^+^ channels were inhibited (Kim, 2021; Kim et al., 2023). We aimed to obtain a broader pharmacological profile of α-Mangostin in K^+^ channels, and we investigated members of all six subfamilies of K_2P_ channels as well as representatives of the K_v_ and K_ir_ channel families. Confirming the previous studies, we observed a strong activation of TREK-1 K_2P_ channels (fold change 10.33 ± 1.64; Figure 1A, Figure 1—figure supplement 1) and inhibition of TRESK K_2P_ channels as well as K_v_1.3 channels by ≈50% and ≈60%, respectively (Figure 1A, B, Figure 1—figure supplement 1). Furthermore, we identified three additional targets of α-Mangostin: strongly inhibited were the K_2P_ members TWIK-1^m^ (≈88%), TWIK-2^m^ (≈50%), and TASK-3 (≈80%) (Figure 1A, B, Figure 1—figure supplement 1). In contrast, BK channels were most potently activated, with a fold change of 20.41 ± 2.39 for BKα channels and of 24.42 ± 3.85 for BKα/β1 channels, which is the complex predominantly present in smooth muscle cells of the vasculature (Figure 1A, C). This suggested that BK channels, especially the BKα/β1 heteromer, could be the molecular target mediating the vasorelaxant Mangostin effects reported in above studies.

**Figure 1—figure supplement 1.**
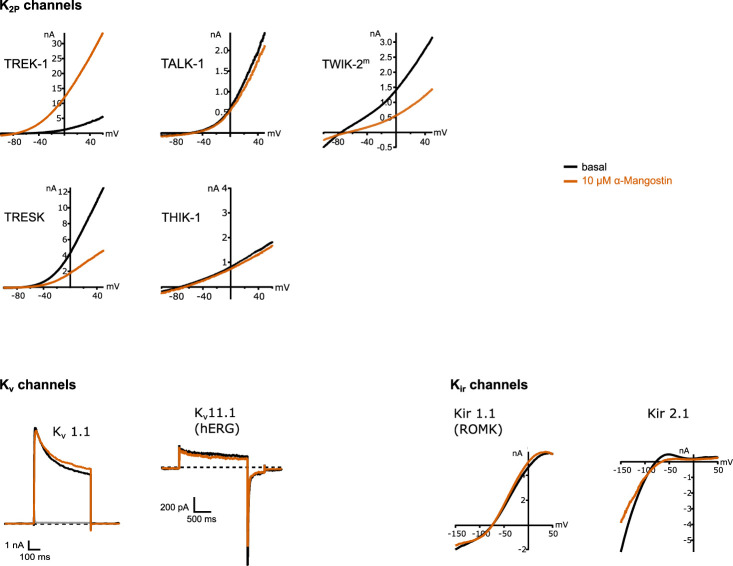
Effect of α-Mangostin on the current of potassium channels from different families. Representative whole-cell current traces recorded from the indicated channels before and after application of 10 µM α-Mangostin. K_2P_ and K_ir_ channels were recorded using a ramp protocol from –100 to +50 mV or –150 to +50 mV, respectively, K_v_1.1 and K_v_1.3 channels were recorded using a rectangle pulse to +40 mV, and hERG was recorded with a rectangle pulse to +60 mV followed by hyperpolarization to –120 mV. All measurements were made in transiently transfected HEK293 cells in physiological potassium gradients with 100 nM intracellular free Ca^2+^. Intracellular K^+^ was exchanged for Rb^+^ for TWIK channels. TWIK-2^m^ denotes channels where the dibasic retrieval motif was removed to enhance membrane expression (I289A/L290A). Data and statistics see Source data file 1.

The preparations from *G. mangostana* plants that are consumed as ‘nutraceuticals’ contain an unstandardized mixture of secondary plant metabolites, among them other Mangostin derivatives. Therefore, we also investigated their modulatory effect on TREK-1, BKα, and BKα/β1 currents (Figure 1D). The very similar γ-Mangostin-activated BKα channels with slightly higher efficacy (fold change of 38.54 ± 4.8) than TREK-1 and BKα/β1 channels (fold change of 11.35 ± 1 and 29.32 ± 5.03). Interestingly, the dietary supplement (solubilized equivalent to 10 µM α-Mangostin) activated TREK1, BKα, and BKα/β1 channels comparably to pure α-/γ-Mangostin (fold change of 9.18 ± 2.74, 19.98 ± 2.99, and 20.92 ± 3.49; Figure 1D), highlighting that mangostane xanthones retain their activity in such extracts.

The application of different α-Mangostin concentrations resulted in a fast and dose-dependent activation of BKα and BKα/β1 channels, with a similar apparent EC_50_ of 3.16 ± 1.13 and 2.57 ± 1.32 µM, suggesting that the potency of α-Mangostin is not affected by the presence of the β1-subunit (Figure 1E). However, we frequently noticed a decline of currents at concentrations >12 µM and limited solubility of higher Mangostin concentrations, which may imply that the actual maximal activation could not be reached.

### α-Mangostin shifts the voltage activation of BKα and BKα/β1 channels to more negative values

Having established the prominent activation of BKα and BKα/β1 channels by α-Mangostin, we wanted to gain more insight into its activation mechanism. The open probability of BK channels is controlled by voltage signals, calcium signals, or shift of the closed–open equilibrium (e.g., by mutations or modulators), and the current further depends on the single-channel amplitude and the time the channel adopts an open conductive state. We therefore investigated these aspects of BK channel gating in macroscopic and single-channel measurements.

**Figure 2.**
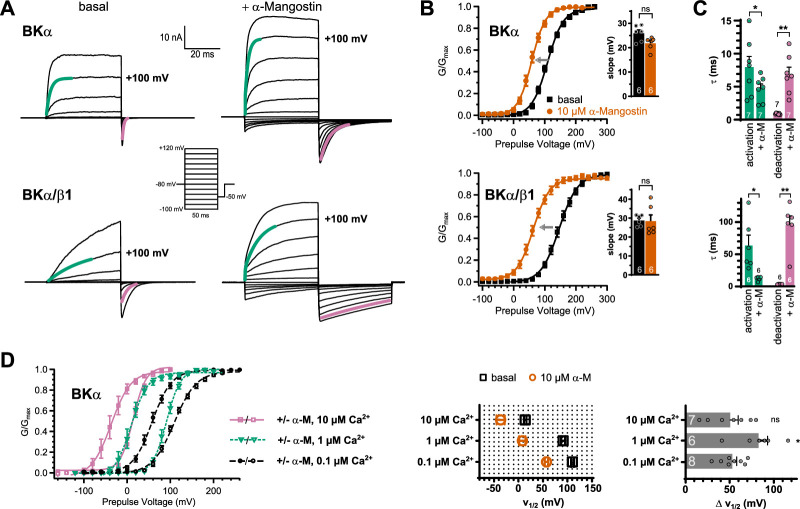
Effects of α-Mangostin on BKα and BKα/β1 channel gating. (**A**) Representative current traces for BKα and BKα/β1 channels before and after activation by 10 µM α-Mangostin. Cells were measured in symmetrical 140 mM bi-ionic conditions with 140 mM Cs^+^ as the intracellular ion and 100 nM free Ca_i_^2+^. Currents were elicited by a family of rectangle pulses from –100 to up to +300 mV in 20 mV increments from a holding potential of –80 mV, followed by repolarization to –50 mV to elicit inward tail currents. (**B**) *GV* relationships for BKα and BKα/β1 channels in the basal state and activated by 10 µM α-Mangostin, derived from tail current analysis of recordings as in (**A**). The gray arrow illustrates the shift of voltage activation toward more negative voltages caused by α-Mangostin and the inset shows the slope ± SEM of the Boltzmann fits. (**C**) Activation and deactivation kinetics of BKα and BKα/β1 channels in the basal state and after activation by 10 µM α-Mangostin. The τ ± SEM of activation/deactivation was determined from exponential fits to the current traces at +100 mV, as shown by the green and purple colored lines in panel (**A**). (**D**) *GV* relationships of BKα channels in different free Ca_i_^2+^ concentrations before and after activation by 10 µM α-Mangostin, derived from tail current analysis as above. Pulse voltages were –100 to +300 mV/repolarization to –50 mV from a holding potential of –80 mV for 100 nM free Ca_i_^2+^, –120 to +200 mV/repolarization to –50 mV from a holding potential of –80 mV for 1 µM free Ca_i_^2+^, and –160 mV to +100 mV/repolarization to –80 mV from a holding potential of –120 mV for 10 µM free Ca_i_^2+^, in symmetrical 140 mM bi-ionic conditions with 140 mM Cs^+^ as the intracellular ion. The *V*_½_ values ± SEM before and after α-Mangostin application and the resulting shifts (Δ*V*_½_ ± SEM) are shown in the bar graphs. Data and statistics see Source data file 1.

As we already observed in the ramp measurements, the threshold of BK voltage activation was shifted to more negative potentials upon α-Mangostin application (Figure 1C). Therefore, we first examined if α-Mangostin affects the voltage activation of BK channels. We used a step protocol over a range of potentials followed by a repolarization step to elicit tail currents in symmetrical bi-ionic conditions where intracellular K^+^ was replaced with Cs^+^ to reduce the strong outward currents in whole-cell experiments (Figure 2A; Cs^+^ does not alter voltage activation Piskorowski and Aldrich, 2006). We quantified the change in voltage activation induced by α-Mangostin by calculating the voltage of half-maximal activation (*V*_½_) from the Boltzmann fit of conductance–voltage (*GV*) relationships (Figure 2B). The application of 10 µM α-Mangostin caused a substantial shift of the *V*_½_ by 53.08 ± 4.9 mV to more negative voltages in BKα channels (from 110.45 ± 2.69 to 57.37 ± 3.6 mV). This effect was even more pronounced in smooth muscle-like BKα/β1 channels, where *V*_½_ shifted by 82.42 ± 4.96 mV to more hyperpolarized voltages (from 147.25 ± 5.66 to 64.83 ± 4.25 mV). The slope of the Boltzmann fit was not different before and after α-Mangostin activation in both channels (Figure 2B, insets). Hence, BKα and BKα/β1 channels already opened at less depolarized voltages in the presence of α-Mangostin, whereas voltage activation itself was not affected.

**Figure 2—figure supplement 1.**
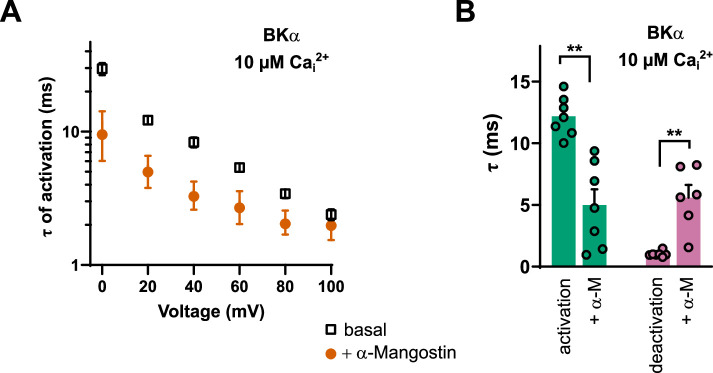
Change of activation and deactivation time course upon α-Mangostin activation of BKα channels measured with 10 µM Ca_i_^2+^. (**A**) Time constant (*τ*) of activation for the voltage range between 0 and +100 mV before and after activation by 10 µM α-Mangostin (*n* = 7). (**B**) Time constants of activation (*n* = 7) and deactivation (*n* = 6) at +20 mV are shown to illustrate their change within the physiological voltage range. Time constants were obtained by fitting a monoexponential function to the current traces after rectangle depolarization (for activation) and after a repolarization step to elicit tail currents (for deactivation) as shown in Figure 2A. Data and statistics see Source data file 1.

### α-Mangostin predominantly affects deactivation of BKα and BKα/β1 channels

An inspection of the currents elicited by the families of rectangle pulses shows that gating kinetics are altered in the α-Mangostin-activated state (Figure 2A). The shift in *V*_½_ toward more negative potentials upon α-Mangostin activation could be caused either by an acceleration of channel activation, or by a slowing down of channel deactivation, or a combination of both. We therefore analyzed the kinetics of activation and deactivation by fitting monoexponential functions to the time course of outward and tail currents to obtain their time constants (*τ*). BKα channels are characterized by fast activating current and a fast deactivation visible as decay of the tail current, while the presence of the β1 subunit slows these kinetics down (Figure 2A, bottom; Dworetzky et al., 1996). After application of 10 µM α-Mangostin, activation of BKα channels was moderately accelerated 1.7-fold at +100 mV, with a *τ* of activation of 7.96 ± 1.64 ms before and 4.71 ± 0.71 ms afterwards (Figure 2C). Additional measurements in 10 µM Ca_i_^2+^ where outward currents are present at lower voltages showed a bigger effect with less depolarization (0–100 mV; Figure 2—figure supplement 1A). The *τ* for deactivation at –50 mV increased ≈7-fold from 0.9 ± 0.04 to 6.85 ± 1.11 ms at +100 mV (Figure 2C; Figure 2—figure supplement 1B). Again, α-Mangostin action was more pronounced in the smooth muscle-like BKα/β1 channels, where activation was more than fivefold faster with *τ* values of 63.76 ± 16.03 ms before and 12.36 ± 1.20 ms after α-Mangostin application. Deactivation kinetics were most affected with a substantial ≈27-fold increase in *τ* from 3.60 ± 0.16 to 95.6 ± 13.99 ms (Figure 2C). These results suggest that the mechanism of α-Mangostin action differentially affects gating transitions that are associated with activation and deactivation.

As BK channels can be massively activated by Ca_i_^2+^ under physiological conditions, we further obtained *GV* relationships in the basal and in the activated state for two higher Ca_i_^2+^ concentrations relevant in smooth muscle cells (Figure 2D). α-Mangostin shifted the V_½_ in 10 µM free Ca_i_^2+^ (representative for a local increase subsequent to a Ca^2+^ spark) by 50.55 ± 9.13 mV to more negative potentials, comparable to the resting state with 0.1 µM free Ca_i_^2+^ (53.08 ± 4.9 mV), and a slightly higher *V*_½_ shift of 82.74 ± 10.17 mV in 1 µM Ca_i_^2+^ (a range more likely in a global Ca_i_^2+^ elevation). Thus, α-Mangostin activates BK channels across a wide range of intracellular Ca²^+^ concentrations, indicating Ca²^+^-independent efficacy.

### α-Mangostin activation mechanism on single-channel and macroscopic current level

**Figure 3.**
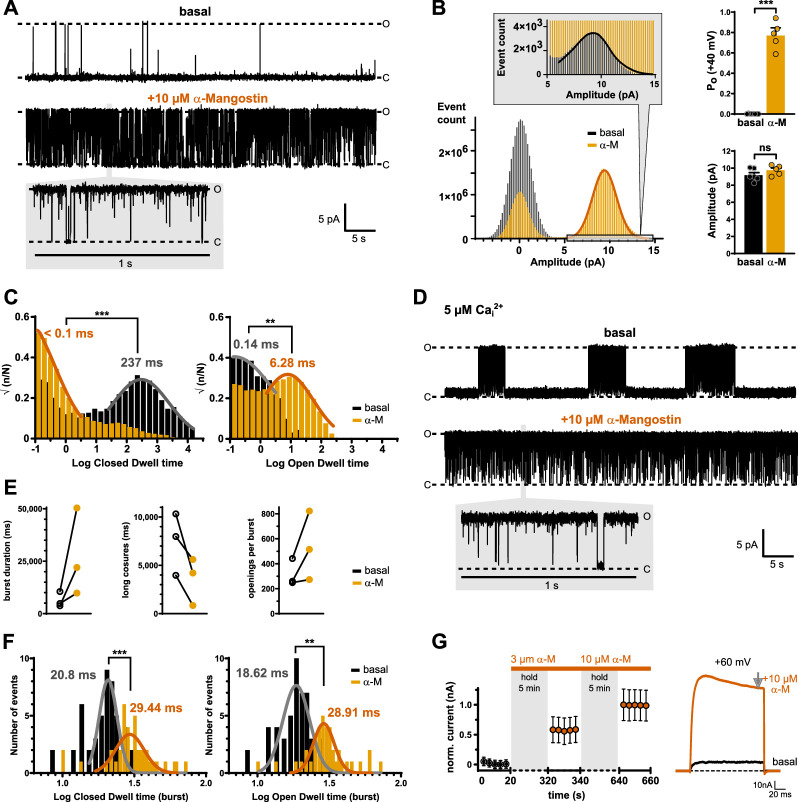
Activation mechanism of α-Mangostin. (**A**) Exemplary current traces of a single BKα channel in the basal state and after activation by 10 µM α-Mangostin (1 min recordings; inset shows 1 s; O and C denote open and closed levels). Single-channel currents were recorded at +40 mV in inside-out patches from transiently transfected HEK293 cells in symmetrical potassium gradients with 100 mM free Ca_i_^2+^ (*n* = 4–7). (**B**) All-points histograms with Gaussian fits for the basal and α-Mangostin-activated state, and bar graphs of the derived mean ± SEM open probabilities (*P*_o_) and amplitudes. The inset magnifies the open peak in the basal state. (**C**) Closed and open dwell time histograms with fits for channels in the basal and in the α-Mangostin-activated state derived after event detection in single-channel measurements as shown in (**A**). (**D**) Exemplary current traces of a single BKα channel recorded as in (**A**), but with 5 µM free Ca_i_^2+^. (**E**) Burst duration, duration of long closed times, and number of openings per burst derived from burst analysis (*n* = 3 patches). (**F**) Closed and open dwell time distribution within bursts with fits for channels in the basal and in the α-Mangostin-activated state. (**G**) Normalized mean ± SEM currents of BKα channels before and after application of 3 or 10 µM α-Mangostin in the closed state. Channels were held closed at –80 mV and only very shortly pulsed to +60 mV after 5 min incubation to assess the current size/activation state of the first and the following pulses. Measurements were done in transiently transfected HEK293 cells in whole-cell mode in physiological potassium gradients with 100 nM Ca_i_^2+^ as shown by the representative current traces to the right, and steady-state currents were analyzed (grey arrow). Data and statistics see Source data file 1.

The marked slowing of deactivation visible as the slow tail current decay in macroscopic current recordings suggests that individual channels are open for longer periods of time in the presence of α-Mangostin. To investigate the effect of α-Mangostin on individual channels, we recorded single-channel currents in excised inside-out patches from HEK293 cells in the presence of 100 nM free Ca_i_^2+^ (Figure 3A). Under these conditions, the open probability (*P*_o_) of BK channels at a potential of +40 mV was very low (0.002 ± 0.0008), but rose to 0.77 ± 0.08 after activation by 10 µM α-Mangostin (Figure 3B) with only very brief closings (Figure 3A, 1 s inset). The single-channel amplitude derived from the all-points-histogram was not different between basal and α-Mangostin-activated states (9.18 ± 0.29 and 9.76 ± 0.26 pA; Figure 3B). We constructed dwell time distributions to determine the differences in the duration that a single channel resides in the closed or open state (Figure 3C). The log closed dwell time distributions showed a large shift of the main component to shorter dwell times, from 2.38 ± 0.23 (237 ms) to –1.39 ± 0.36 (less than 0.1 ms). The open dwell time distribution revealed that the log open time was markedly reduced from –0.84 ± 0.45 to 0.8 ± 0.24 (i.e., from 0.14 to 6.28 ms).

BK channels are known to show bursting gating behavior (Geng and Magleby, 2014). As bursts did not occur in our measurements in resting conditions, we elevated free Ca_i_^2+^ to be able to perform burst analysis (Figure 3D). Mean burst duration was increased and the long closures between bursts were shortened, explaining the increase in overall open probability (Figure 3E). The intraburst kinetic distributions showed that α-Mangostin increased intraburst closed time from 20.8 ± 1.2 to 29.4 ± 1.3 ms, thereby reducing very brief closures (flicker), indicating that transitions became slower (Figure 3F). The higher open probability within bursts resulted from both prolonged opening (as open dwell times increased from 18.6 ± 1.2 to 28.9 ± 1.2 ms) and from an increased probability of reopening (visible as increased number of openings per burst; Figure 3E), consistent with an open-state stabilization that explains the reduced deactivation seen in macroscopic recordings.

The fact that the open and closed times were both affected in the overall dwell time distributions as well as in intraburst distributions suggests that α-Mangostin can bind to the open and the closed state. Accordingly, application of α-Mangostin on closed BKα channels (at –80 mV) resulted in maximal activation with the first depolarization pulse, indicating that α-Mangostin reached its site of action during the closed period (Figure 3G).

### Localization of the α-Mangostin binding site

We recently described the polypharmacology of the class of negatively charged activators (NCAs) in different potassium channels, specifically in TREK-1 and BK channels. The BK channel opener GoSlo-SR-5-6 (Roy et al., 2012; Webb et al., 2015) also activated TREK-1 channels, while BL-1249 known as TREK/TRAAK channel opener, also activated BK channels. We identified their common binding site in the pore close to the fenestration of TREK-1 channels and molecular dynamics (MD) simulations predicted the equivalent binding site in the pore of BK channels (Schewe et al., 2019). The question arose if α-Mangostin could also occupy this binding site, as in part suggested in a docking by Kim et al., 2023, who proposed that P183 and L304, which both are part of the NCA binding site, interact with α-Mangostin in TREK-1 channels. Therefore, we measured the dose-dependent α-Mangostin activation of TREK-1 wildtype channels and its ‘signature’ mutant of the fenestration, L304C. As expected, the apparent affinity is markedly reduced in the L304C mutant in the measurable concentration range (Figure 4—figure supplement 1A). Quaternary ammonium ions are known to occupy the central cavity of K^+^ channels as TREK-1 and BK just below the SF near the NCA binding site (Li and Aldrich, 2004; Piechotta et al., 2011; Fan et al., 2024). In a competition experiment, we recorded dose–response relationships and determined the IC_50_ value for tetrapentylammonium (TPA) with and without preactivation by 10 µM α-Mangostin. The TPA IC_50_ value increased ≈9-fold from 0.72 ± 0.64 to 6.33 ± 0.86 µM in the presence of α-Mangostin (Figure 4—figure supplement 1B), showing that α-Mangostin was present in the pore and hindered the access of TPA to its binding site. Finally, cysteine scanning mutagenesis of residues in M2 and M4 revealed that mainly G181, I182, P182 on M2 and G308 in M4 were involved, while most M4 residues tested had intermediary effects (Figure 4—figure supplement 1C), suggesting that the binding region of α-Mangostin between M2 and M4 overlaps with the NCA binding site identified with the help of BL-1249, but is not completely identical (Figure 4—figure supplement 1D).

**Figure 4.**
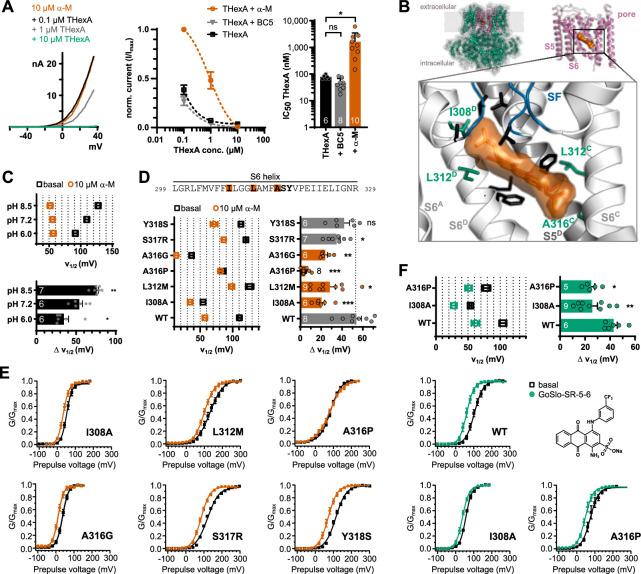
Investigation of the binding site of α-Mangostin in BKα channels. (**A**) Competition experiment showing a reduction of the block caused by THexA in the presence of α-Mangostin. Left, representative current traces for different THexA concentrations in the presence of 10 µM α-Mangostin; middle, competition experiment analysis showing the relative current of BKα channels in different THexA concentrations in the absence and in the presence of 10 µM α-Mangostin or 100 µM BC5, which does not bind in the pore; and right, estimated IC_50_ values of THexA alone and in the presence of α-Mangostin or BC5. Whole-cell currents were recorded from transiently transfected HEK293 cells with a ramp protocol (–100 to +50 mV) in a physiological potassium gradient with 100 nM free Ca_i_^2+^ and data are shown as mean ± SEM at +40 mV. (**B**) Molecular docking of α-Mangostin to the human BK channel structure (PDB ID 6v3g, Ca^2+^-free state). The full-length structure (green) was reduced to the inner pore region (pink) for the docking, and the zoom-in shows the best pose for α-Mangostin with interacting residues in stick representation; green residues mark hits from the following functional assay. Protein chain B was removed for clarity. See Figure 4—figure supplement 3 for the Ca^2+^-bound state. (**C**) Voltage of half-maximal activation (*V*_½_) before and after activation by 10 µM α-Mangostin in different pH and the resulting shifts in *V*_½_ (Δ*V*_½_). The pH was changed intra- and extracellularly. (**D**) Voltage of half-maximal activation (*V*_½_) before and after activation by 10 µM α-Mangostin, and the resulting shifts in *V*_½_ (Δ*V*_½_). (**E**) *GV* relationships for the six BKα mutants in the S6 segment. (**F**) Voltage of half-maximal activation (*V*_½_) before and after activation by 1 µM GoSlo-SR-5-6, the resulting shifts in *V*_½_ (Δ*V*_½_), and the *GV* relationships for the wildtype and two BKα mutants. *GV* relationships were measured as in Figure 2, and all data represent mean ± SEM. Data and statistics see Source data file 1.

We next probed if α-Mangostin accesses the pore cavity of BKα channels, as in TREK channels, or binds elsewhere in the protein. We conducted a competition experiment as above and measured the dose-dependent inhibition by 0.1, 1, and 10 µM tetrahexylammonium (THexA; Figure 4A). In the presence of α-Mangostin, the inhibition by THexA was clearly reduced, and the apparent THexA affinity (as estimated IC_50_) was steeply decreased ≈21-fold from 77.51 ± 5.53 nM to 1.64 ± 0.58 µM, showing that the presence of α-Mangostin hindered the access of THexA to its binding site. This is consistent with previous findings that GoSlo-RS-5-6, which binds in the BK pore, also competes with THexA (Schewe et al., 2019). In contrast, the presence of BC5, an activator shown to bind at the interface between the transmembrane and intracellular domains (Zhang et al., 2022), did not interfere with THexA binding (estimated THexA IC_50_ 45.60 ± 8.05 nM; Figure 4A).

**Figure 4—figure supplement 1.**
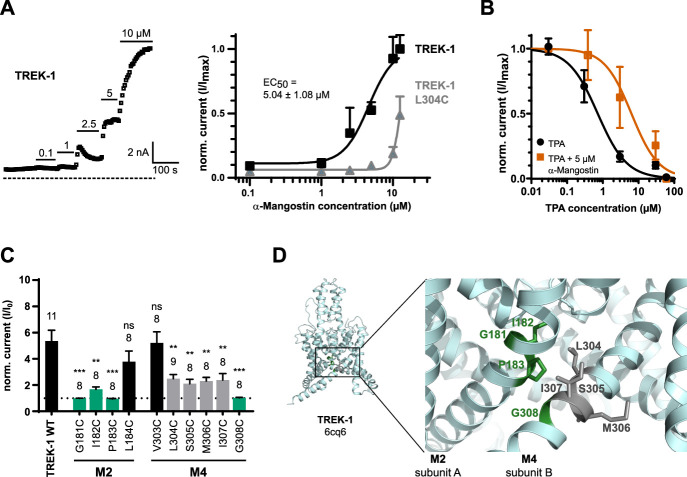
Activation of TREK-1 channels by α-Mangostin and investigation of the binding region. (**A**) Dose-dependent activation of TREK-1 channels (left) and the derived dose–response relationship for the WT and for the L304C mutant located at the fenestration entrance. (**B**) Dose–response relationship for TPA in the absence or presence of 5 µM α-Mangostin (left) and the IC_50_ values derived from Hill fits (right). (**C**) Cysteine scanning mutagenesis of M2 and M4 residues belonging to the fenestration site revealed the fold change of their currents after application of 5 µM α-Mangostin. (**D**) Location of the hit residues (green) in the TREK-1 crystallographic structure (PDB ID 6cq6). All measurements were done in transiently transfected HEK293 cells in physiological potassium gradients with a ramp protocol from –100 to +50 mV (holding potential –80 mV) and currents were analyzed at +40 mV. Data and statistics see Source data file 1.

**Figure 4—figure supplement 2.**
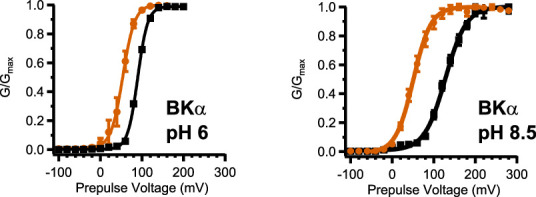
Activation of BKα channels by 10 µM α-Mangostin in different pH. *GV* relationships for BKα channels in the basal state and activated by 10 µM α-Mangostin, derived from tail current analysis. HEK 293 cells were measured in whole-cell configuration in symmetrical 140 mM bi-ionic conditions with 140 mM Cs^+^ as the intracellular ion and 100 nM free Ca_i_^2+^. Currents were elicited by a family of rectangle pulses from –100 to up to +300 mV in 20 mV increments from a holding potential of –80 mV, followed by repolarization to –50 mV to elicit inward tail currents. The pH was changed intra- and extracellularly. Data and statistics see Source data file 1.

**Figure 4—figure supplement 3.**
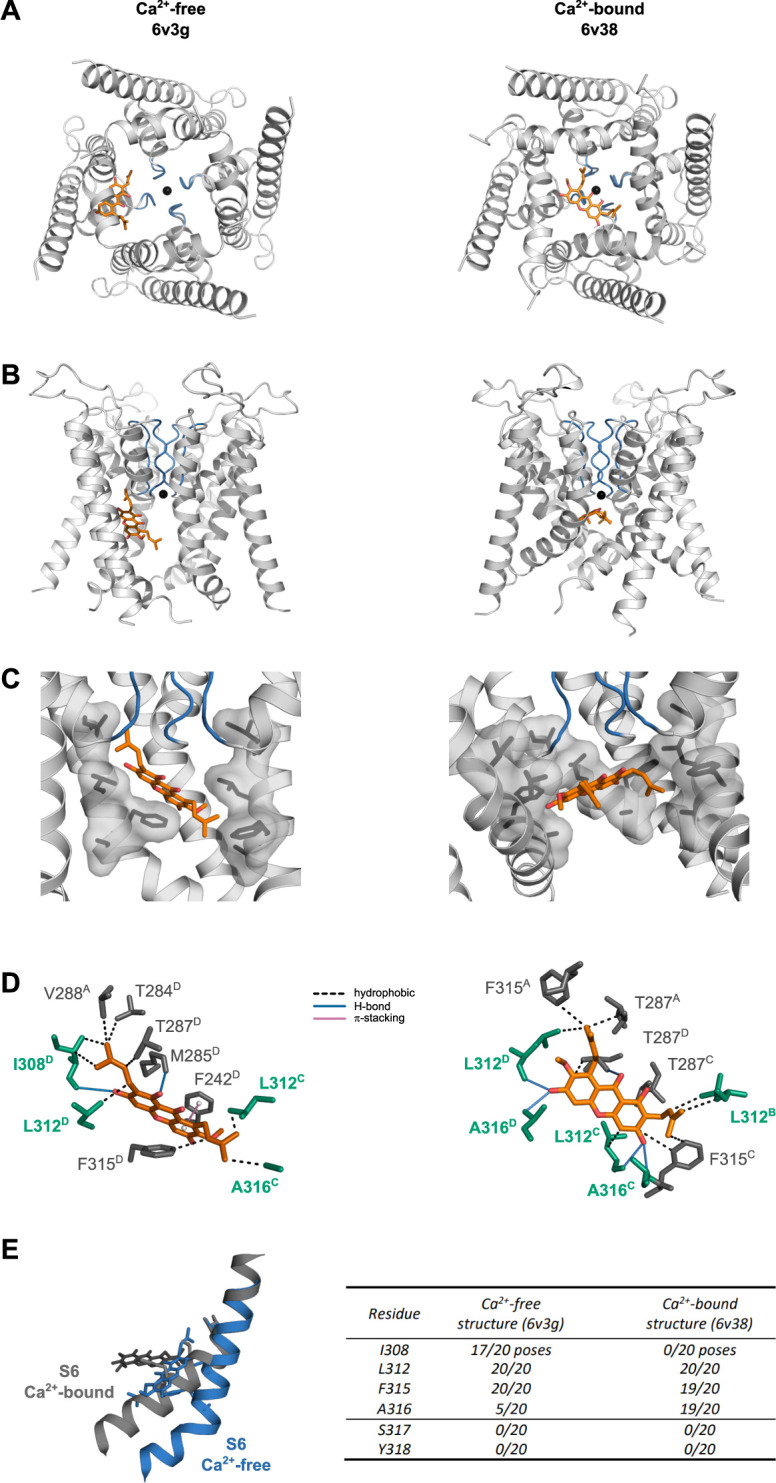
Molecular docking of α-Mangostin to the Ca^2+^-free and Ca^2+^-bound human BKα channel. Central pore region of the Ca^2+^-free (PDB ID 6v3g) and the Ca^2+^-bound structure (PDB ID 6v38) comprised of S5, pore helices, and S6 segments with the best pose of α-Mangostin, viewed from the intracellular side (**A**) and the membrane plane (**B**). (**C**) Visualization of the binding pocket comprising S6 segments (chain B removed for clarity), and (**D**) potentially interacting residues in stick representation with the mutants exhibiting a loss in the shift of voltage activation upon α-Mangostin application highlighted in green. (**E**) Overlay of the S6 helices in the Ca^2+^-free (blue) and the Ca^2+^-bound state (grey) illustrating the upwards movement that concomitantly shifts α-Mangostin to a more horizontal pose, and a table comparing the interactions in both states.

At least one hydroxyl group of α-Mangostin is predicted to be negatively charged in physiological pH (National Center for Biotechnology Information, 2025); therefore, we tested the impact of solution pH on the activation potency. Indeed, the shift in *V*_½_ induced by 10 µM α-Mangostin was pH-dependent (Figure 4C, Figure 4—figure supplement 2). In pH 6, the shift was reduced to 34.71 ± 5.63 mV, while it increased to 75.97 ± 2.02 mV in pH 8.5, indicating that the negative charge may be critical for effective activation and α-Mangostin might resemble an NCA-like compound.

In addition, we used molecular docking to determine the location of a possible binding site in the BKα inner pore in the Ca^2+^-bound (presumably open) and the Ca^2+^-free (presumably closed) structure (Figure 4B, Figure 4—figure supplement 3). We analyzed 20 poses which were all located at the cavity wall in middle S6 between residues I308 and V319, in a pocket below the SF, without obstructing the central passageway for K^+^ ions (Figure 4—figure supplement 3A). In the Ca^2+^-free and Ca^2+^-bound structures, three and five poses were clustered with the lowest binding energies of –8.58 and –8.64 kcal mol^–1^, respectively. For the best pose in the Ca^2+^-free state, possible molecular interactions were predicted for the residues I308, L312, F315, and A316, and α-Mangostin was wedged between S6 segments of adjacent helices, while in the Ca^2+^-bound state where S6 moves upwards, I308 was buried and interactions shifted more to A316, resulting in a more horizontal molecule position (Figure 4—figure supplement 3C, E).

To functionally investigate the predicted binding site, we mutated residues in the pore-lining S6 helix with side chains facing into the cavity. Many substitutions in this region affect gating of BK channels and are involved in intersubunit S6–S6 contact (Wu et al., 2009; Zhou et al., 2011). Therefore, we chose only substitutions that were most WT-like with respect to Ca_i_^2+^ and voltage sensitivity, that is, I308A, L312M, A316P for the hits from the docking, and additionally S317R and Y318S as internal controls that were not predicted to be part of the binding site (Chen et al., 2014). We first assessed the shift in voltage activation induced by 10 µM α-Mangostin (Figure 4D, E). *V*_1/2_ shifts of Y318S and S317R were not or only moderately different compared to the wildtype channel (41.46 ± 5.17 and 34.33 ± 3.37 mV). In contrast, the three mutants I308A, L312M, and in particular A316P had a markedly reduced shift in *V*_1/2_ (19.97 ± 3.12, 27.89 ± 5.42, and 4.56 ± 1.23 mV, respectively). To ensure that the almost absent shift in A316P was not caused by a general disruption of allosteric signal transduction, we also included A316G, which showed a reduction in *V*_1/2_ comparable to I308A and L312M (23.57 ± 2.05 mV).

Furthermore, we tested GoSlo-SR-5-6 as an alternative activator, which binds in the same pore region involving A316, and was shown to also compete with THexA (Schewe et al., 2019; Zhang et al., 2022). Like for α-Mangostin, I308A and A316P reduced the shift in *V*_½_ induced by 1 µM GoSlo-SR-5-6 from 42.71 ± 3.05 mV to 25.89 ± 4.05 and 25.0 ± 2.8 mV; however, A316P did not abolish GoSlo-SR-5-6 activation, showing that the complete loss of activation for α-Mangostin was likely caused primarily by a loss of binding and not by an interference of the mutation with the drug transduction mechanism (Figure 4F). Hence, we conclude that α-Mangostin binds to and activates BKα channels via the upper S6 segment, critically involving residues I308, L312, and A316.

### α-Mangostin activation in BK–Ca_v_ nanodomains

**Figure 5.**
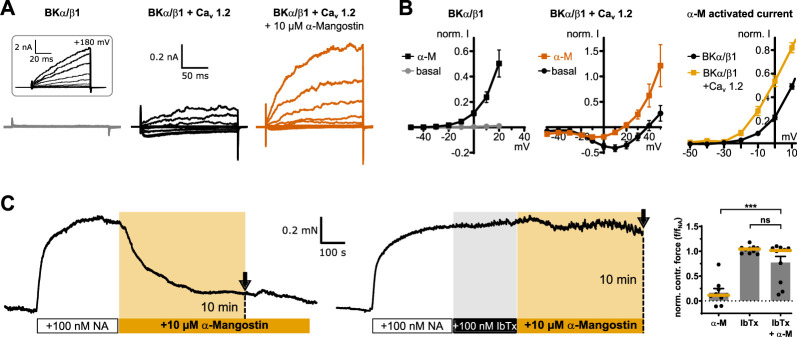
α-Mangostin activation of BK channels in physiological settings. (**A**) Representative whole-cell current traces of BKα/β1 channels alone and BKα/β1 coexpressed with Ca_v_1.2 channels before and after application of 10 µM α-Mangostin. Currents were measured in Ca_i_^2+^-free conditions in a physiological potassium gradient with a family of voltage steps from –50 to +50 mV in 10 mV increments. The inset shows voltage activation with a family protocol up to +200 mV to show the presence of BKα/β1 channels. (**B**) Currents of BKα/β1 channels and BKα/β1–Ca_v_ complexes before and after application of 10 µM α-Mangostin plotted against voltage. The last panel shows the α-Mangostin-activated currents for the range –50 to 10 mV obtained by subtracting the current before α-Mangostin application from the current after application for each potential (mean ± SEM, *n* = 8–11 for each condition). (**C**) Representative contraction force recordings of aortic preparations from mice. 10 µM α-Mangostin were either applied directly to aortic preparations precontracted with 100 nM Noradrenaline (NA; left), or the precontracted preparations were incubated with 100 nM Iberiotoxin (IbTx) before α-Mangostin application (right). The contraction force was analyzed 10 min after α-Mangostin addition (dotted lines in recordings). The bar graph shows the normalized contraction force of preparations as mean ± SEM together with the median (orange). Data and statistics see Source data file 1. DMSO controls are shown in Figure 5—figure supplement 1.

In resting smooth muscle cells, there is no BK channel activation within their physiological voltage range. However, in native tissue, BK channels are organized in nanodomains together with Ca_v_ channels that can generate an increase in Ca_i_^2+^ concentration in the range of several orders of magnitude in their vicinity, allowing BK channels to open (Shah et al., 2021). This BK–Ca_v_ complex can be restored in heterologous expression systems, and, as the intracellular solution contains 5 mM EGTA to suppress an overall increase of Ca_i_^2+^, BK channels will activate only when nearby Ca_v_ channels cause a high local Ca_i_^2+^ concentration (Berkefeld et al., 2006; Berkefeld and Fakler, 2008). BKα/β1 channels expressed alone therefore produced virtually no current upon stepwise depolarization to +50 mV (Figure 5A, B), but could be activated by α-Mangostin (Figure 5A, B). In BKα/β1–Ca_v_ complexes, Ca^2+^ inward currents were elicited, which allowed BKα/β1 channels to open in a more negative voltage range, and these BKα/β1 currents were further enhanced by 10 µM α-Mangostin (Figure 5A, B). The α-Mangostin-activated current (as difference between BKα/β1-Ca_v_ currents before and after activation) was activated at more negative potentials when BKα/β1 and Ca_v_ channels were coexpressed than when BKα/β1 channels were expressed alone (Figure 5B). Hence, α-Mangostin could potentiate BKα/β1 channel currents upon local Ca_i_^2+^ increase through nearby Ca_v_-channels, as it could occur upon a Ca_i_^2+^ spike in a cell.

**Figure 5—figure supplement 1.**
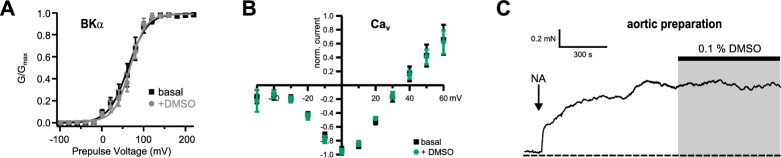
Vehicle control for BKα and Ca_v_1.2 channels and aortic tissue. (**A**) *GV* relationships for BKα channels with and without DMSO, derived from tail current analysis (mean ± SEM; *n* = 4). (**B**) Current–voltage relationship of Ca_v_1.2 channels with and without DMSO (mean ± SEM; *n* = 3). HEK 293 cells were measured in whole-cell configuration in symmetrical 140 mM bi-ionic conditions with 140 mM Cs^+^ as the intracellular ion and 100 nM free Ca_i_^2+^. Currents were elicited from a holding potential of –80 mV by a family of rectangle pulses from –100 to +240 mV followed by repolarization to –20 mV to elicit inward tail currents for BKα channels, and by a family of rectangle pulses from –50 to +60 mV for Ca_v_ channels. Data and statistics see Source data file 1. (**C**) Representative measurement showing the application of 0.1% DMSO to precontracted mouse aortic tissue in the organ bath.

### α-Mangostin relaxes vascular tissue in aortic preparations via BK channels

Cardiovascular benefits of Mangostins could arise from vasodilatory effects leading to a reduction of blood pressure. Years ago, a study reported that γ-Mangostin, in which the methoxy group of α-Mangostin is exchanged for a hydroxyl group, induced relaxation of rat aortic rings, but no molecular target was found (Tep-Areenan and Suksamrarn, 2012). We sought to demonstrate that Mangostin-induced vasorelaxation of native vascular tissue is indeed mediated by BK channels. Aortic preparations from mice were equilibrated at a contraction force of 2–3 mN and subsequently precontracted half-maximally with 100 nM noradrenaline. The application of 10 µM α-Mangostin quickly and efficiently induced relaxation, while relaxation was absent or greatly attenuated when the specific BK channel inhibitor Iberiotoxin (IbTx) was applied for 5–6 min before α-Mangostin was administered (Figure 5C). In total, α-Mangostin reduced the normalized contraction force of the preparations by more than 85% (to 0.16 ± 0.08) compared to the precontracted state, while it did not change after IbTx application alone (1.04 ± 0.002) or after preblock of BK channels (0.78 ± 0.12), proving that BK channels must mediate the vasodilative effect seen upon Mangostin treatment.

## Discussion

We provide mechanistic insight into how a natural xanthone compound modulates BK channels, which are major players in vascular function. α-Mangostin activates BK channels by shifting their voltage sensitivity and slowing deactivation, with lesser effects on activation and marked effects on Ca_i_^2+^ sensitivity. We show that binding of α-Mangostin is state-independent, and we provide evidence for a binding site in the gating-sensitive S6 segment.

### Mangostin binds to closed and open channels and promotes the open state

BK channel p_o_ is controlled by Ca_i_^2+^, voltage, a shift of the closed–open equilibrium of the pore domain, or a combination of those stimuli (Cui et al., 1997). Mangostin shifted the voltage activation curve to more negative potentials, meaning an increased open probability at negative voltages. The slope of the *GV* relationship was not changed neither in homomeric BKα nor in the smooth muscle BKα/β1 channels, indicating that voltage sensitivity itself was not directly affected by α-Mangostin. Shifts in the *V*_½_ values induced by α-Mangostin were present over a range of Ca_i_^2+^ concentrations relevant in smooth muscle cells (0.1, 1, and 10 µM), which implies that the closed–open equilibrium is shifted toward the open state by a mechanism distinct from Ca_i_^2+^ sensing, or that at least the Ca_i_^2+^ sensing mechanism is not likely to contribute significantly to α-Mangostin activation.

The activation can be mainly explained by the marked slowing of the deactivation time constants. Consistently, the mean open dwell time was increased in the single-channel measurements and brief closings within bursts were reduced, while we observed no change in amplitude with and without α-Mangostin (corresponding to a conductance of ≈240 pS). The impact on open as well as closed dwell time distributions and the instantaneous activation of macroscopic currents from the closed state suggest that the molecule binds state-independently.

In the presence of the β1 subunit, as in vascular BK channels (Knaus et al., 1994), the activation characteristics were enhanced. The activation time course was little affected in BKα channels, but in BKα/β1 channels an acceleration of the activation caused by α-Mangostin became more prominent, counteracting the slow activation kinetics brought by the β1 subunit (McManus et al., 1995). Additionally, the slow deactivation time course in the presence of the β1 subunit was further slowed down remarkably. As the affinity (as estimated EC_50_) was very similar and the binding site is located in the S6 segment in the inner pore, the stronger shift in the *V*_½_ value is likely not due to changes in binding, but reflects the enhanced shift of the closed–open equilibrium toward the open state conferred by the β1 subunit. Such β-subunit-dependent effects have also been reported for other modulators, for example, DHS-I (McManus et al., 1993; Giangiacomo et al., 1998), DHA (Hoshi et al., 2013a), arachidonic acid (Martín et al., 2021), 17β-estradiol (Valverde et al., 1999), and some GoSlo compounds (Large et al., 2015).

### The binding site of α-Mangostin includes residues in the gating-sensitive S6 segment

We demonstrated the presence of α-Mangostin in the channel pore by competition for binding with quaternary ammonium ions known to bind below the SF (Li and Aldrich, 2004; Fan et al., 2024), and predicted by molecular docking to the Ca^2+^-free and the Ca^2+^-bound state, identified residues in the S6 segment that are part of the binding site.

α-Mangostin binds in the cleft formed by the S6 helices of adjacent subunits, and substitution of the nonpolar residues I308, L312, and especially A316 most strongly reduced the shift in *V*_½_ induced by α-Mangostin. All residues are known to participate in gating: they are part of a hydrophobic network within S6 that acts as a hinge upon channel opening, and polar substitutions strongly favor the open state (Zhou et al., 2011; Chen et al., 2014; Hite et al., 2017). L312 has further been shown to stabilize intersubunit interaction with F315, whose destruction opens the channel (Wu et al., 2009). In the open state, S6 is thought to move upwards, as seen in the Ca^2+^-bound cryo-EM structure (Kallure et al., 2023; Yamanouchi et al., 2023; Redhardt et al., 2024), thereby burying the I308 sidechain and diminishing the dimension of the upper cleft. Therefore, the contribution of I308 to α-Mangostin activation is likely allosteric in nature, and the pose of the molecule changes with the S6 position.

The α-Mangostin binding region overlaps with the reported binding site of the NCA GoSlo-SR-5-6, where L312, A316, S317, and V319 are involved (Webb et al., 2015; Schewe et al., 2019), but it is not identical. When we functionally investigated the α-Mangostin binding site close to the fenestration in TREK-1 channels, we also found only a partial overlap with key residues involved in the NCA binding site. Interaction with P183 and L304 was consistent with an earlier molecular docking (Kim et al., 2023), but the strongest reduction of activation was found for residues in M2 rather than M4.

### Mechanism of mangostin activation

We expect that small structural or electrostatic changes in this gating-sensitive region strongly affect the closed–open equilibrium (Wu et al., 2009; Zhou et al., 2011; Chen et al., 2014). Previous studies suggest several alternative mechanisms by which α-Mangostin could induce channel opening. We have shown that NCAs bind to a region below the SF in several K^+^ channels, such as K_2P_ (e.g., TREK), hERG, or BK channels to induce channel opening (Schewe et al., 2019). We speculated that the negative charge common to these activators might alter SF ion occupancy via an electrostatic mechanism, thereby promoting SF opening. Our finding that the potency of α-Mangostin depends on pH suggests that a negative charge may also be critical for its activation mechanism. However, the importance of the SF for BK gating remains unresolved. Furthermore, α-Mangostin also did not increase the single-channel conductance as seen for NCAs in TREK-2 channels, which are known to be gated at the SF (Schewe et al., 2019). Therefore, the contribution of the SF to the Mangostin effect requires further investigation. Alternatively, α-Mangostin binding to the gating-critical S6 region could induce gating at a putative lower gate; however, such a gate is currently speculative, as the available BK structures lack clear evidence for a lower gate (but the final closed state may still be elusive) (Hite et al., 2017; Tao et al., 2017).

In addition to the concepts of an SF gate or a lower gate, hydrophobic gating has been proposed as an alternative gating mechanism in BK channels. MD simulations suggest that the pore of metal-free (presumably closed) BK structures undergoes dewetting, whereas the pore of metal-bound (presumably open) structures remains hydrated (Jia et al., 2018; Gu and de Groot, 2023). Thus, binding of negatively charged α-Mangostin might prevent or antagonize dewetting of the hydrophobic cavity and thereby stabilize the conductive pore state, probably with substantial involvement of I308. Indeed, a recent MD study suggested that the NCA NS11021, a smooth muscle relaxant with a biarylthiourea structure, can enter the dewetted BK pore to promote hydration (Rockman et al., 2020; Nordquist et al., 2024). However, the same MD simulations also indicated that NS11021 has no stable binding pose but engages in various hydrophobic interactions with pore-cavity residues. In contrast, our results suggest a more stable binding pose with specific interactions to a number of residues in the Ca^2+^-free as well as in the Ca^2+^-bound state, as inferred from our mutagenesis and docking data. Clearly, further work is required to elucidate the mechanism of BK channel gating and the exact way Mangostin and other NCAs modulate this process to promote channel activation.

### Activation of BK channels in nanodomains and relaxation of aortic tissue

Our data demonstrate that α-Mangostin is able to potentiate Ca_v_-induced currents, and that α-Mangostin-activated BK channels are responsible for the relaxation of mouse vascular tissue in a conceptual ex vivo experiment, providing a very probable explanation for the reduction of blood pressure reported in a recent animal study (Xu et al., 2024).

We reconstructed nanodomains of BK and Ca_v_1.2 channels, as for example present in arterioles (Berkefeld et al., 2006; Tykocki et al., 2017). In smooth muscle cells, BK activation is strictly coupled to changes in Ca_i_^2+^. Physiological Ca_i_^2+^ concentrations lie between 100 nM in the resting state, 0.4–1 µM upon activation of a Ca^2+^ mobilizing receptor (Savineau and Marthan, 2000; Hill-Eubanks et al., 2011), and up to several ten micromolar (10–40 µM) when sparks form locally (Zhuge et al., 2002; Berkefeld et al., 2010). Transient outward currents through activated BK channels then act as negative feedback, repolarize the cell, and terminate Ca^2+^ influx (Eisvand et al., 2022). We demonstrated that the α-Mangostin-activated current is potentiated by the coupling to Ca_v_ channels. BKα/β1 currents in the physiological voltage range of smooth muscle cells (−50 to –30 mV) would be very small in resting Ca_i_^2+^, while the p_o_ would be already very high when exceeding 10 µM Ca^2+^, leaving less room for activation. α-Mangostin would particularly impact potentiation of BK currents upon physiological Ca^2+^ elevation (i.e., between 1 and 10 µM) as well as in the physiological voltage range, and would synergistically lower the BKα/β1 activation threshold to enhance the negative feedback mechanism.

Accordingly, our investigation of precontracted aortic tissue from mice showed a remarkable and robust relaxation after application of α-Mangostin. Such relaxation of aortic rings after application of α- or γ-Mangostin was reported before (Chairungsrilerd et al., 1996; Chairungsrilerd et al., 1997; Tep-Areenan and Suksamrarn, 2012), but the exact molecular target remained inconsistent. We did not assess the effects of Mangostin on other components of the contraction pathway, such as Cav channels or ryanodine receptors, and therefore cannot exclude their modulation. However, given the robust activation of BK channels and the complete loss of relaxation following preincubation with the selective BK inhibitor iberiotoxin, our data indicate that BK channels represent the primary molecular target of Mangostins in vascular smooth muscle cells.

Interestingly, relaxation was also reported for other xanthone derivatives (Cheng and Kang, 1997; Wang et al., 2002; Capettini et al., 2009; Câmara et al., 2010; Diniz et al., 2013), which legitimizes further research of substituted xanthones as pharmacophore. Robust data on bioavailability and the existence and nature of active metabolites are currently lacking. A small study claimed that α-Mangostin was bioavailable in humans after supplement ingestion (Kondo et al., 2009). Animal research analyzed metabolites and showed that maximal plasma concentrations are reached 1 hr after oral intake in mice (Ramaiya et al., 2012; Petiwala et al., 2014; Han et al., 2015). However, no metabolites have been functionally investigated to help in optimization of the Mangostin pharmacophore; however, our finding that γ-Mangostin has a slightly higher potency in activating TREK-1 and BKα channels may be a starting point.

Our screen across different K^+^ channel families revealed that other representatives of the K_2P_ (TWIK-1 and TASK-3) were particularly inhibited by more than 60%. This could also link other claimed benefits of Mangostin to their molecular targets. Importantly, hERG currents were unaffected, mitigating the cardiotoxicity risk upon consumption of Mangostin nutraceuticals. BK channel function is decreased in chronic conditions that are often associated with the lack of a balanced diet and age, such as metabolic syndrome, diabetes, and obesity (Tykocki et al., 2017). Consequences such as increased vascular tone and hypertension, ultimately leading to reduced cardiovascular health and premature death, may therefore be addressed with Mangostin xanthones as a potential new class of BK channel activators.

## Materials and methods

**Key resources table:** 

Reagent type (species) or resource	Designation	Source or reference	Identifiers	Additional information
Gene (*Homo sapiens*)	*KCNK2*	GenBank	NM_001017425.2	hTREK-1b
Gene (*Homo sapiens*)	*KCNK16*	GenBank	NM_032115.3	hTALK-1
Gene (*Homo sapiens*)	*KCNK18*	GenBank	NM_181840.1	hTRESK
Gene (*Homo sapiens*)	*KCNK1*	GenBank; Feliciangeli et al., 2010	NM_002245.3	hTWIK-1^m^; I293A, I294A
Gene (*Homo sapiens*)	*KCNK6*	GenBank; Bobak et al., 2017	NM_004823.1	hTWIK-2^m^; I289A, L290A
Gene (*Homo sapiens*)	*KCNK9*	GenBank	NM_001282534.1	hTASK-3
Gene (*Homo sapiens*)	*KCNK13*	GenBank	NM_022054.3	hTHIK-1
Gene (*Mus musculus*)	*Kcnma1*	GenBank	NM_001014797.3	mBKα (Slo1.1); G/S-rich N-terminus removed, corresponding to residues 65–1236 of the native channel
Gene (*Mus musculus*)	*Kcnmb1*	GenBank	NM_031169.4	mβ1
Gene (*Homo sapiens*)	*KCNA1*	GenBank	NM_000217.3	hK_v_ 1.1
Gene (*Homo sapiens*)	*KCNA3*	GenBank	NM_002232.5	hK_v_ 1.3
Gene (*Homo sapiens*)	*KCNH2*	GenBank	AJ512214.1	hK_v_ 11.1 (hERG1b)
Gene (*Homo sapiens*)	*KCNJ1*	GenBank	NM_000220.4	hK_ir_ 1.1 (ROMK)
Gene (*Mus musculus*)	*KCNJ2*	GenBank	NM_008425.4	mK_ir_ 2.1
Gene (*Rattus norvegicus*)	*Cacna1c*	GenBank	M67515.1	Ca_v_α 1C
Gene (*Rattus norvegicus*)	*Cacnb1*	GenBank	NM_017346.1	Ca_v_ β1
Gene (*Rattus norvegicus*)	*Cacna2d1*	GenBank	AF286488	Ca_v_ α2δ1
Gene (synthetic)	*EYFP*	Addgene	https://www.addgene.org/vector-database/2688/	pEYFP
Strain, strain background (*Escherichia coli*)	DH5α	NEB	Cat. #: C2987	Chemically competent cells
Strain, strain background (*Mus musculus*)	CD1/CHR2 Mice (1 female, 7 male)	IMSR	RRID:IMSR_CRL:022	age 5–6 months
Cell line (*Homo sapiens*)	HEK293	Sigma-Aldrich/Cellosaurus	RRID:CVCL_0045	
Cell line (*Cricetulus griseus*)	CHO-K1	Sigma-Aldrich/Cellosaurus	RRID:CVCL_0214	
Peptide, recombinant protein	Iberiotoxin	Alomone Labs (Jerusalem, Israel)	Cat. #: STI-400	
Commercial assay or kit	Lipofectamine 2000	Invitrogen, Thermo Fisher, Schwerte, Germany	Cat. #: 11668019	
Commercial assay or kit	FuGENE	Promega, Walldorf, Germany	Cat. #: E2311	
Commercial assay or kit	Venor GEM OneStep	Minerva Biolabs	Cat. #: 11-8025	Mycoplasm PCR test
Chemical compound, drug	α-Mangostin	Merck (Darmstadt, Germany)	Cat. #: M3824	
Chemical compound, drug	γ-Mangostin	Merck (Darmstadt, Germany)	Cat. #: M6824	
Chemical compound, drug	Mangosteen antioxidant support	https://Swanson.com	Cat. #: SWH259	
Chemical compound, drug	BC-5	MP Biomedicals (Irvine, USA)	Arg-4-methoxy-2-naphthylamine	
Chemical compound, drug	TPA	Merck (Darmstadt, Germany)	Cat. #: 241970	
Chemical compound, drug	THexA	Merck (Darmstadt, Germany)	Cat. #: 263834	
Chemical compound, drug	GoSlo-SR-5-6	Courtesy of Mark Hollywood (Dundalk Institute of Technology)	Sodium 1-amino-4-((3trifluoromethylphenyl)amino)-9,10-dioxo-9,10-dihydroanthracene-2-sulfonate	
Software, algorithm	Patchmaster v2.78	HEKA Elektronik (Lambrecht, Germany)	RRID:SCR_000034	
Software, algorithm	Fitmaster v2.92	HEKA Elektronik (Lambrecht, Germany)	RRID:SCR_016233	
Software, algorithm	PyMOL	Schrodinger LLC, 2015	RRID:SCR_000305	
Software, algorithm	AutoDock4 v4.2.6/MGL Tools v1.5.7	Morris et al., 2009	RRID:SCR_012746	
Software, algorithm	LabChart	AD Instruments, Mannheim, Germany	RRID:SCR_018833	
Software, algorithm	IgorPro	WaveMetrics, Portland, USA	RRID:SCR_000325	
Software, algorithm	pClamp Clampfit v11.2.2.17	Molecular Devices, San Jose, USA	RRID:SCR_011323	
Other	EPC10	HEKA Elektronik (Lambrecht, Germany)	RRID:SCR_018399	

### Substances

α-Mangostin, γ-Mangostin, TPA, THexA, MgATP, NaGTP, and noradrenaline were purchased from Merck (Darmstadt, Germany). BC5 (Arg-4-methoxy-2-naphthylamine) was obtained from MP Biomedicals (Irvine, USA), and Iberiotoxin from Alomone Labs (Jerusalem, Israel). GoSlo-SR-5-6 (sodium 1-amino-4-((3trifluoromethylphenyl)amino)-9,10-dioxo-9,10-dihydroanthracene-2-sulfonate) was provided by Mark Hollywood. Mangostane food supplement stating a content of 50 mg α-Mangostin per capsule was ordered online (https://Swanson.com). Aiming for a 10 mM stock solution with respect to α-Mangostin, 205 mg of the powder found in the capsules was dispersed in 5 ml DMSO, brought into solution by vortexing and ultrasonication, and filtered through a 0.45 µM PTFE membrane to remove remaining particulate matter. TPA, THexA, noradrenaline, BC5, and Iberiotoxin were prepared as 1:1000 stocks in H_2_O, and the Mangostins and GoSlo-RS-5-6 in DMSO at 50 mM. Aliquots were stored at –20°C and diluted to the final concentration in extracellular solution. The final DMSO concentration did not exceed 0.025%.

### Molecular biology, cell culture, and transfection

Coding sequences for channels and subunits listed in Table 1 were subcloned in pcDNA3.1 (https://www.addgene.org/vector-database/2097/) or pFAW vectors containing a CMV promoter for expression in cultured cells, or in the pBF *Xenopus* oocyte vector for low expression in the single- channel experiments (see below). Amino acid substitutions were introduced by site-directed mutagenesis PCR according to the QuikChange protocol (Stratagene, La Jolla, USA) with custom primers containing the desired base exchange. All constructs were verified by Sanger sequencing. To enhance plasma membrane expression, the retrieval motif was removed in hTWIK-1 and hTWIK-2 channels by the substitutions I293A, I294A (Feliciangeli et al., 2010) and I289A, L290A (Bobak et al., 2017), respectively, yielding hTWIK-1^m^ and hTWIK-2**^m^** channels.

**Table 1.** Channels and subunits used in this study.

Name	Organism	Gene name	Genbank Acc. No.
hTREK-1b	*Homo sapiens*	*KCNK2*	NM_001017425.2
hTALK-1	*Homo sapiens*	*KCNK16*	NM_032115.3
hTRESK	*Homo sapiens*	*KCNK18*	NM_181840.1
hTWIK-1^m^	*Homo sapiens*	*KCNK1*	NM_002245.3
hTWIK-2^m^	*Homo sapiens*	*KCNK6*	NM_004823.1
hTASK-3	*Homo sapiens*	*KCNK9*	NM_001282534.1
hTHIK-1	*Homo sapiens*	*KCNK13*	NM_022054.3
mBKα (Slo1.1)^*^	*Mus musculus*	*Kcnma1*	NM_001014797.3
mβ1	*Mus musculus*	*Kcnmb1*	NM_031169.4
hK_v_ 1.1	*Homo sapiens*	*KCNA1*	NM_000217.3
hK_v_ 1.3	*Homo sapiens*	*KCNA3*	NM_002232.5
hK_v_ 11.1 (hERG1b)	*Homo sapiens*	*KCNH2*	AJ512214.1
hK_ir_ 1.1 (ROMK)	*Homo sapiens*	*KCNJ1*	NM_000220.4
mK_ir_ 2.1	*Mus musculus*	*KCNJ2*	NM_008425.4
Ca_v_ 1.2:			
Ca_v_α 1C	*Rattus norvegicus*	*Cacna1c*	M67515.1
Ca_v_ β1	*Rattus norvegicus*	*Cacnb1*	NM_017346.1
Ca_v_ α2δ1	*Rattus norvegicus*	*Cacna2d1*	AF286488

* G/S-rich N-terminus removed, corresponding to residues 65–1236 of the native channel.

HEK293 cells (RRID:CVCL_0045, Sigma-Aldrich) were cultivated in Dulbecco’s modified Eagle’s medium supplemented with 10% FCS and penicillin/streptomycin (100 U ml^–1^/100 µg ml^–1^) in 5% CO_2_ at 37°C. For cultivation of CHO-K1 cells (RRID:CVCL_0214, Sigma-Aldrich), the medium was additionally supplemented with non-essential amino acid mix (Gibco MEM-NEAA; Thermo Fisher, Schwerte, Germany) and 10 mM HEPES. Cell lines were regularly tested for mycoplasm contamination by PCR (Venor GEM OneStep, Minerva Biolabs) and cell identity was visually controlled by comparison to the ATCC database.

HEK293 cells were transiently transfected with Lipofectamine 2000 (Invitrogen, Thermo Fisher, Schwerte, Germany) or FuGENE (Promega, Walldorf, Germany) according to the manufacturer’s instructions and incubated overnight. pEYFP (https://www.addgene.org/vector-database/2688/) was included as a transfection marker. In BKα/β1 coexpressions, the β1 subunit was used in 10-fold excess to ensure a uniform channel population. Cells were trypsinized and seeded onto glass coverslips 2–3 hr prior to electrophysiological experiments.

### Electrophysiology

Voltage clamp experiments were conducted with a HEKA EPC10 amplifier (RRID:SCR_018399) controlled by Patchmaster Software (v2.78; RRID:SCR_000034; HEKA Elektronik, Lambrecht, Germany). Standard measurements were done in transiently transfected HEK293 cells in the whole-cell configuration in physiological potassium gradients. Pipettes were pulled from thin-walled borosilicate glass, fire-polished, and had resistances of 1.4–2 MΩ. Series resistance was compensated to at least 70%. The extracellular solution contained (in mM): 135 NaCl, 5 KCl, 2 CaCl_2_, 2 MgCl_2_, 10 D(+)-glucose, 10 HEPES (pH 7.3). The intracellular solution was (in mM): 140 KCl, 2 MgCl_2_, 1 CaCl_2_, 2.5 EGTA, 10 HEPES (pH 7.3), corresponding to ≈100 nM free Ca^2+^. For K_ir_ 1.1 and 2.1 channel recordings, 3 mM MgATP and 0.3 mM NaGTP were included. Where indicated, K^+^ was substituted for Cs^+^ to reduce the outward currents of BK channels. Cells were held at –80 mV and a voltage ramp from –100 to +60 mV of 1 s duration was applied every 5 s. For K_v_ channels, a rectangle pulse was used as indicated in the figure legends.

The activation of BK channels was measured as conductance *G* in symmetrical potassium solutions to ensure a unitary conductance across the voltage range. Cs^+^ was used as the intracellular ion to reduce outward currents. The extracellular solution contained (in mM): 140 KCl, 2 CaCl_2_, 2 MgCl_2_, 10 D(+)-glucose, 10 HEPES (pH 7.3). The intracellular solution was (in mM): 140 CsCl, 2 MgCl_2_, 1 CaCl_2_, 2.5 EGTA, 10 HEPES (pH 7.3), corresponding to ≈100 nM free Ca^2+^ (calculated with WEBMAXC). For recordings with 1 µM and 10 µM free Ca_i_^2+^, CaCl_2_ and EGTA were raised to 2/2.2 and 2/4.04 mM, respectively. Cells were held at negative holding potentials and currents were elicited with a family of rectangle voltage pulses in 20 mV increments to positive potentials as needed for saturation of the tail currents, followed by a repolarization step to elicit the tail currents as detailed in the figure legends. The currents were analyzed with Fitmaster (HEKA Elektronik, Lambrecht, Germany; RRID:SCR_016233), and conductance–voltage (*GV*) relationships were generated from normalized tail currents and fit to a standard Boltzmann relationship to obtain *V*_½_ values. Individual fits were then averaged for mean *V*_½_ values.

$$\displaystyle \frac{G}{G_{max}}\mathrm{=}\frac{1}{1\mathrm{+}e^{\frac{\left (V\mathrm{-}V_{1/2}\right)}{k}}}$$

where *G*_max_ is the maximal tail current, *V*_½_ is the voltage of half-maximal activation of the current, and *k* is the slope factor.

Activation in different pH was measured using the following solutions: Extracellular solution pH 8.5 contained (in mM): 140 KCl, 2 CaCl_2_, 2 MgCl_2_, 10 D(+)-glucose, 10 Ampso; intracellular solution pH 8.5 contained (in mM): 140 CsCl_2_, 10 Ampso, 2 MgCl_2_, 1 CaCl_2_, 2.05 EGTA. Extracellular solution pH 6 was (in mM): 140 KCl, 2 CaCl_2_, 2 MgCl_2_, 10 D(+)-glucose, 10 MES; intracellular solution pH 6 contained (in mM): 140 CsCl_2_, 10 MES, 2 MgCl_2_, 0.5 CaCl_2_, 11 EDTA. The pH was changed intra- and extracellularly.

In TPA competition experiments for TREK-1 channels, a dose–response relationship in the absence and presence of α-Mangostin was recorded and the IC_50_ was obtained from a Hill fit. The THexA competition experiments for BKα channels were conducted as 3-point determinations using 0.1 and 1 µM THexA as concentrations close to half-maximal inhibition without and with α-Mangostin and 10 µM THexA for full block. To estimate the IC_50_, a simplified 4-parameter logistic regression was used, assuming *a*=0 and *b*=1.

$$\displaystyle y\mathrm{=}d\mathrm{+}\frac{a\mathrm{-}d}{1\mathrm{+}\left (\frac{x}{c}\right)^{b}}\text{}\rightarrow \text{}c\mathrm{=}\frac{x}{\left (\frac{d}{y}\mathrm{-}1\right)}$$

where *a*: lower asymptote, *b*: slope, *c*: IC_50_, *d*: upper asymptote, *x*: THexA concentration, and *y*: current.

### Single-channel recordings

Single channels were recorded at room temperature from excised inside-out patches of HEK293 cells transiently transfected with BKα in a continuous voltage protocol at +40 mV. To increase the chances of obtaining patches with only one channel, a pBF vector with a suboptimal beta globin promoter yielding only low expression was used. Pipette resistances were 12–15 MΩ, and symmetrical intra- and extracellular solution contained (in mM) 140 KCl, 2 MgCl_2_, 1 CaCl_2_, 2.5 EGTA, 10 HEPES (pH 7.3 with KOH), corresponding to ≈100 nM free Ca^2+^, or 140 KCl, 2 MgCl_2_, 2.36 CaCl_2_, 2.4 EGTA, 10 HEPES (pH 7.3 with KOH), corresponding to ≈5 µM free Ca^2+^. Currents were recorded at a sampling rate of 100 kHz with a final bandwidth $f_{c}$ of 7.4 kHz using an EPC10 amplifier (RRID:SCR_018399) controlled by Patchmaster software (v2.78; RRID:SCR_000034; HEKA Elektronik, Lambrecht, Germany; RRID:SCR_000034). Traces were analyzed in Clampfit (v11.2.2.17; RRID:SCR_011323; Molecular Devices, San Jose, USA). The filter rise time $T_{r}\mathrm{=}\frac{0.3321}{f_{c}}$ was 45 µs, and openings >2 $T_{r}$ were fitted (Colquhoun and Sigworth, 1995). Histograms were calculated in Clampfit and fitted with a Gaussian function to obtain amplitudes and dwell times using Prism (v8.4.3; GraphPad Software Inc, San Diego, USA; RRID:SCR_002798). Single-channel conductance was calculated from $g\mathrm{=}\frac{I}{\left (V_{m}\mathrm{-}E_{K^{\mathrm{+}}}\right)}$, where *g*, conductance; *I*, single-channel current; *V*_*m*_, membrane voltage; *E*_K+_, K^+^ equilibrium potential. Bursts were detected using a minimum closed interval of 200 ms. Bursts with a single opening (0 ms intraburst interval) were included in the calculation of mean closed times within a burst. Means were calculated per patch. The fits of kinetic distribution data are descriptive as data was pooled.

### Reconstitution of nanodomains

BKα/β1–Ca_v_ complexes were restored as described by Berkefeld et al., 2006 with the following modification: For nanodomain formation of Ca_v_ 1.2 and BKα/β1, CHO-K1 cells were seeded onto coverslips and microinjected (InjectMan 4, Eppendorf, Hamburg, Germany) with a DNA mixture of Ca_v_α 1C, Ca_v_ β1, Ca_v_ α2δ1, BKα, β1, and EYFP in the ratio 10:10:10:10:100:1 to ensure the presence of all subunits. Cells were incubated overnight, and currents were recorded from EYFP-positive cells 14–18 hr after injection. Cells were measured in a physiological potassium gradient with a voltage step protocol from –50 to +60 mV in 10 mV increments. The extracellular solution contained (in mM): 135 NaCl, 5 KCl, 2 CaCl_2_, 2 MgCl_2_, 10 D(+)-glucose, 10 HEPES (pH 7.3). The intracellular solution was (in mM): 140 KCl, 2 MgCl_2_, 5 EGTA, 10 HEPES (pH 7.3). α-Mangostin was added to the bath solution at a final concentration of 10 µM.

### Molecular docking

The input structures for molecular docking were generated by truncating the structure of the human BKα channel in the Ca^2+^-free and Ca^2+^-bound state (PDB ID 6v3g, 6v38) to only the pore region (res T229-R329) using PyMOL (Schrodinger LLC, 2015; RRID:SCR_000305). Docking was performed with AutoDock4 (v4.2.6; RRID:SCR_012746) using MGL Tools (v1.5.7) (Morris et al., 2009). A run with lower resolution grid was done, and after α-Mangostin was seen in the pore, the docking was repeated with a gridbox restricted to the cavity below the SF. Clustering and interactions were analyzed with the built-in functions of MGL Tools.

### Aortic smooth muscle preparations

Animal care and use and the procedures to obtain aortic tissue from mice were licensed and performed according to German animal protection law, the regulations of the state authorities of Mecklenburg-West Pomerania, and the institutional standards of the University of Rostock (Tierschutz-G_Nr. A414-18). 1 female and 7 male CD1/CHR2 mice (RRID:IMSR_CRL:022) at the age of 5–6 months were randomly chosen and killed by decapitation following isoflurane-narcosis, and the aorta was removed. Inclusion/exclusion criteria did not apply, and all animals were used for further study. The aortic wall was dissected, and tissue strips of ≈15 × 2 mm were obtained using the spiral cut technique (Peiper and Schmidt, 1972). Aortic preparations were tethered to glass holders and transferred to temperature-controlled 37°C bath chambers filled with Krebs solution (pH 7.4; in mM: 112 NaCl, 4.7 KCl, 2.5 CaCl_2_, 1.2 MgCl_2_, 1.2 KH_2_PO_4_, 25 NaHCO_3_, and 11.5 glucose). The bath solution was bubbled with 95% O_2_/5% CO_2_. The contraction force was recorded with mechanoelectric transducers (Fort100, WPI, Sarasota, USA), connected to a PowerLab controlled by LabChart (AD Instruments, Mannheim, Germany; RRID:SCR_018833). A prestrain of 2.5–3.9 mN was applied, depending on the thickness of preparations, and strips equilibrated for ca. 45 min. The NA concentration necessary to achieve half-maximal contraction was determined by measuring a dose–response relationship, and 100 nM NA were subsequently used for precontraction. After a plateau was reached (2–4 min), α-Mangostin and IbTx were diluted into the bath solution to their final concentrations of 10 µM (final DMSO concentration 0.1%) and 100 nM, respectively, and the contraction force was recorded for ca. 20 min. Recordings were analyzed with LabChart.

### Data analysis and statistics

All data are given as mean ± SEM and the number of data points (cells or patches) is given above the bars or in the figure legends. Mean data underlying figures is reported in Source data file 1. Measurements were made from at least two different transfections. Mean currents were analyzed with Patchmaster (HEKA Elektronik, Lambrecht, Germany; RRID:SCR_000034). Fitmaster (HEKA Elektronik, Lambrecht, Germany; RRID:SCR_016233) was used to generate *GV* relationships and fits with the Boltzmann function, and for the monoexponential fits to obtain activation and deactivation time constants. EC_50_/IC_50_ values were obtained by fitting a Hill equation to the dose–response data using IgorPro (v6.3.7; WaveMetrics, Portland, USA; RRID:SCR_000325) or Prism (v8.4.3; GraphPad Software Inc, San Diego, USA; RRID:SCR_002798).

$$\displaystyle I_{0}\mathrm{+}\frac{I_{max}\mathrm{-}I_{0}}{1\mathrm{+}\left (\frac{c_{1/2}}{c}\right)^{h}}$$

with *I*_0_, basal current; *I*_max_, activated current; *c*, concentration; *c*_½_, EC_50_/IC_50_; *h*, Hill coefficient.

Statistical analysis was conducted with the built-in functions of Prism. If data were not normally distributed, nonparametric tests were used. Variances were compared with *F*-tests. Two means were then compared with a paired or unpaired two-tailed *t*-test. Three or more groups were compared using one-way ANOVA when variances and standard deviation were considered equal by Bartlett’s test; otherwise, the Brown–Forsythe and Welch ANOVA was applied. Dunnett’s T3 post hoc test was used to account for multiple comparisons in ANOVA, and Dunn’s post hoc test was used for nonparametric tests. Statistical tests and p values are reported in Source data file 1. Symbols for p values are used in the figures as follows: ***p < 0.001, **p ≤ 0.01, *p ≤ 0.05, ns: p ≥ 0.05.

Structures were visualized with PyMOL (Schrodinger LLC, 2015); RRID:SCR_000305 and figures were assembled with Inkscape v1.3 (GNU General Public License, v3; RRID:SCR_014479).

## Data Availability

All mean data and statistical tests shown in figures are included as tables in source data file 1. Source data of electrophysiological measurements and molecular dockings are available from Dryad (https://doi.org/10.5061/dryad.x69p8d00j). The following dataset was generated: CordeiroS PatejdlR BaukrowitzT MusinszkiMA 2026Data from: Natural xanthones as α-Mangostin induce vasorelaxation involving key gating residues in the S6 domain of BK channelsDryad Digital Repository10.5061/dryad.x69p8d00jPMC1314882442089408

## Additional files

MDAR checklist

Source data 1. Summary of data used in figures and applied statistical tests with p values.

## References

[bib1] Alpha-Tocopherol, Beta Carotene Cancer Prevention Study Group (1994). The effect of vitamin E and beta carotene on the incidence of lung cancer and other cancers in male smokers. New England Journal of Medicine.

[bib2] Barenco-Marins TS, Seara FAC, Ponte CG, Nascimento JHM (2025). Pulmonary circulation under pressure: pathophysiological and therapeutic implications of BK channel. Cardiovascular Drugs and Therapy.

[bib3] Bentzen BH, Olesen SP, Rønn LCB, Grunnet M (2014). BK channel activators and their therapeutic perspectives. Frontiers in Physiology.

[bib4] Berkefeld H, Sailer CA, Bildl W, Rohde V, Thumfart J-O, Eble S, Klugbauer N, Reisinger E, Bischofberger J, Oliver D, Knaus H-G, Schulte U, Fakler B (2006). BKCa-Cav channel complexes mediate rapid and localized Ca2+-activated K+ signaling. Science.

[bib5] Berkefeld H, Fakler B (2008). Repolarizing responses of BKCa-Cav complexes are distinctly shaped by their Cav subunits. The Journal of Neuroscience.

[bib6] Berkefeld H, Fakler B, Schulte U (2010). Ca2+-activated K+ channels: from protein complexes to function. Physiological Reviews.

[bib7] Bobak N, Feliciangeli S, Chen C-C, Ben Soussia I, Bittner S, Pagnotta S, Ruck T, Biel M, Wahl-Schott C, Grimm C, Meuth SG, Lesage F (2017). Recombinant tandem of pore-domains in a Weakly Inward rectifying K^+^ channel 2 (TWIK2) forms active lysosomal channels. Scientific Reports.

[bib8] Brenner R, Peréz GJ, Bonev AD, Eckman DM, Kosek JC, Wiler SW, Patterson AJ, Nelson MT, Aldrich RW (2000). Vasoregulation by the beta1 subunit of the calcium-activated potassium channel. Nature.

[bib9] Brown K, Theofanous D, Britton RG, Aburido G, Pepper C, Sri Undru S, Howells L (2024). Resveratrol for the Management of human health: how far have we come? a systematic review of resveratrol clinical trials to highlight gaps and opportunities. International Journal of Molecular Sciences.

[bib10] Câmara DV, Lemos VS, Santos MH, Nagem TJ, Cortes SF (2010). Mechanism of the vasodilator effect of Euxanthone in rat small mesenteric arteries. Phytomedicine.

[bib11] Capettini LS, Campos LVA, Dos Santos MH, Nagem TJ, Lemos VS, Cortes SF (2009). Vasodilator and antioxidant effect of xanthones isolated from Brazilian medicinal plants. Planta Medica.

[bib12] Chairungsrilerd N, Furukawa K, Ohta T, Nozoe S, Ohizumi Y (1996). Pharmacological properties of alpha-mangostin, a novel histamine H1 receptor antagonist. European Journal of Pharmacology.

[bib13] Chairungsrilerd N, Furukawa KI, Ohta T, Nozoe S, Ohizumi Y (1997). γ-Mangostin, a novel type of 5-hydroxytryptamine 2A receptor antagonist. Naunyn-Schmiedeberg’s Archives of Pharmacology.

[bib14] Chen X, Yan J, Aldrich RW (2014). BK channel opening involves side-chain reorientation of multiple deep-pore residues. PNAS.

[bib15] Cheng YW, Kang JJ (1997). Mechanism of vasorelaxation of thoracic aorta caused by xanthone. European Journal of Pharmacology.

[bib16] Colquhoun D, Sigworth FJ, Sakmann B, Neher E (1995). Single-Channel Recording.

[bib17] Cui J, Cox DH, Aldrich RW (1997). Intrinsic voltage dependence and Ca2+ regulation of mslo large conductance Ca-activated K+ channels. The Journal of General Physiology.

[bib18] Diniz TF, Pereira AC, Capettini LSA, Santos MH, Nagem TJ, Lemos VS, Cortes SF (2013). Mechanism of the vasodilator effect of mono-oxygenated xanthones: a structure-activity relationship study. Planta Medica.

[bib19] Di Pierro F, Khan A, Iqtadar S, Mumtaz SU, Chaudhry MNA, Bertuccioli A, Derosa G, Maffioli P, Togni S, Riva A, Allegrini P, Recchia M, Zerbinati N (2022). Quercetin as a possible complementary agent for early-stage COVID-19: Concluding results of a randomized clinical trial. Frontiers in Pharmacology.

[bib20] Dudem S, Large RJ, Kulkarni S, McClafferty H, Tikhonova IG, Sergeant GP, Thornbury KD, Shipston MJ, Perrino BA, Hollywood MA (2020). LINGO1 is a regulatory subunit of large conductance, Ca ^2+^ -activated potassium channels. PNAS.

[bib21] Dworetzky SI, Boissard CG, Lum-Ragan JT, McKay MC, Post-Munson DJ, Trojnacki JT, Chang CP, Gribkoff VK (1996). Phenotypic alteration of a human BK (hSlo) channel by hSlobeta subunit coexpression: changes in blocker sensitivity, activation/relaxation and inactivation kinetics, and protein kinase A modulation. The Journal of Neuroscience.

[bib22] Eisvand F, Imenshahidi M, Ghasemzadeh Rahbardar M, Tabatabaei Yazdi SA, Rameshrad M, Razavi BM, Hosseinzadeh H (2022). Cardioprotective effects of alpha-mangostin on doxorubicin-induced cardiotoxicity in rats. Phytotherapy Research.

[bib23] Fan C, Flood E, Sukomon N, Agarwal S, Allen TW, Nimigean CM (2024). Calcium-gated potassium channel blockade via membrane-facing fenestrations. Nature Chemical Biology.

[bib24] Feliciangeli S, Tardy MP, Sandoz G, Chatelain FC, Warth R, Barhanin J, Bendahhou S, Lesage F (2010). Potassium channel silencing by constitutive endocytosis and intracellular sequestration. The Journal of Biological Chemistry.

[bib25] Ferraguto C, Piquemal-Lagoueillat M, Lemaire V, Moreau MM, Trazzi S, Uguagliati B, Ciani E, Bertrand SS, Louette E, Bontempi B, Pietropaolo S (2024). Therapeutic efficacy of the BKCa channel opener chlorzoxazone in a mouse model of Fragile X syndrome. Neuropsychopharmacology.

[bib26] Geng Y, Magleby KL (2014). Single-channel kinetics of BK (Slo1) channels. Frontiers in Physiology.

[bib27] Giangiacomo KM, Kamassah A, Harris G, McManus OB (1998). Mechanism of maxi-K channel activation by dehydrosoyasaponin-I. The Journal of General Physiology.

[bib28] Gierten J, Ficker E, Bloehs R, Schlömer K, Kathöfer S, Scholz E, Zitron E, Kiesecker C, Bauer A, Becker R, Katus HA, Karle CA, Thomas D (2008). Regulation of two-pore-domain (K2P) potassium leak channels by the tyrosine kinase inhibitor genistein. British Journal of Pharmacology.

[bib29] Gonzalez-Perez V, Lingle CJ (2019). Regulation of BK channels by beta and gamma subunits. Annual Review of Physiology.

[bib30] Gonzalez-Sanabria N, Contreras GF, Rojas M, Duarte Y, Gonzalez-Nilo FD, Perozo E, Latorre R (2025). The BK channel-NS1619 agonist complex reveals molecular insights on allosteric activation gating. bioRxiv.

[bib31] Gu R-X, de Groot BL (2023). Central cavity dehydration as a gating mechanism of potassium channels. Nature Communications.

[bib32] Han SY, You BH, Kim YC, Chin Y-W, Choi YH (2015). Dose-independent ADME properties and tentative identification of metabolites of α-mangostin from garcinia mangostana in mice by automated microsampling and UPLC-MS/MS methods. PLOS ONE.

[bib33] Hanner M, Schmalhofer WA, Munujos P, Knaus HG, Kaczorowski GJ, Garcia ML (1997). The beta subunit of the high-conductance calcium-activated potassium channel contributes to the high-affinity receptor for charybdotoxin. PNAS.

[bib34] Hennekens CH, Buring JE, Manson JE, Stampfer M, Rosner B, Cook NR, Belanger C, LaMotte F, Gaziano JM, Ridker PM, Willett W, Peto R (1996). Lack of effect of long-term supplementation with beta carotene on the incidence of malignant neoplasms and cardiovascular disease. The New England Journal of Medicine.

[bib35] Hill-Eubanks DC, Werner ME, Heppner TJ, Nelson MT (2011). Calcium signaling in smooth muscle. Cold Spring Harbor Perspectives in Biology.

[bib36] Hite RK, Tao X, MacKinnon R (2017). Structural basis for gating the high-conductance Ca2+-activated K+ channel. Nature.

[bib37] Horrigan FT, Aldrich RW (2002). Coupling between voltage sensor activation, Ca2+ binding and channel opening in large conductance (BK) potassium channels. The Journal of General Physiology.

[bib38] Hoshi T, Tian Y, Xu R, Heinemann SH, Hou S (2013a). Mechanism of the modulation of BK potassium channel complexes with different auxiliary subunit compositions by the omega-3 fatty acid DHA. PNAS.

[bib39] Hoshi T, Xu R, Hou S, Heinemann SH, Tian Y (2013b). A point mutation in the human Slo1 channel that impairs its sensitivity to omega-3 docosahexaenoic acid. The Journal of General Physiology.

[bib40] Hoshi T, Heinemann SH (2016). Modulation of BK channels by small endogenous molecules and pharmaceutical channel openers. International Review of Neurobiology.

[bib41] Jang M, Cai L, Udeani GO, Slowing KV, Thomas CF, Beecher CW, Fong HH, Farnsworth NR, Kinghorn AD, Mehta RG, Moon RC, Pezzuto JM (1997). Cancer chemopreventive activity of resveratrol, a natural product derived from grapes. Science.

[bib42] Jia Z, Yazdani M, Zhang G, Cui J, Chen J (2018). Hydrophobic gating in BK channels. Nature Communications.

[bib43] John OD, Mushunje AT, Surugau N, Guad RM (2022). The metabolic and molecular mechanisms of α‑mangostin in cardiometabolic disorders (Review). International Journal of Molecular Medicine.

[bib44] Kallure GS, Pal K, Zhou Y, Lingle CJ, Chowdhury S (2023). High-Resolution Structures Illuminate Key Principles Underlying Voltage and LRRC26 Regulation of Slo1 Channels. bioRxiv.

[bib45] Kim EJ, Kang D, Han J (2011). Baicalein and wogonin are activators of rat TREK-2 two-pore domain K+ channel. Acta Physiologica.

[bib46] Kim HJ (2021). Inhibitory effects of α-Mangostin on T cell cytokine secretion via ORAI1 calcium channel and K+ channels inhibition. PeerJ.

[bib47] Kim SE, Yin MZ, Roh JW, Kim HJ, Choi SW, Wainger BJ, Kim WK, Kim SJ, Nam JH (2023). Multi-target modulation of ion channels underlying the analgesic effects of α-mangostin in dorsal root ganglion neurons. Phytomedicine.

[bib48] Knaus HG, Garcia-Calvo M, Kaczorowski GJ, Garcia ML (1994). Subunit composition of the high conductance calcium-activated potassium channel from smooth muscle, a representative of the mSlo and slowpoke family of potassium channels. The Journal of Biological Chemistry.

[bib49] Kondo M, Zhang L, Ji H, Kou Y, Ou B (2009). Bioavailability and antioxidant effects of a xanthone-rich Mangosteen (Garcinia mangostana) product in humans. Journal of Agricultural and Food Chemistry.

[bib50] Kong C, Jia L, Jia J (2022). γ-mangostin attenuates amyloid-β42-induced neuroinflammation and oxidative stress in microglia-like BV2 cells via the mitogen-activated protein kinases signaling pathway. European Journal of Pharmacology.

[bib51] Large RJ, Kshatri A, Webb TI, Roy S, Akande A, Bradley E, Sergeant GP, Thornbury KD, McHale NG, Hollywood MA (2015). Effects of the novel BK (KCa 1.1) channel opener GoSlo-SR-5-130 are dependent on the presence of BKβ subunits. British Journal of Pharmacology.

[bib52] Latorre R, Castillo K, Carrasquel-Ursulaez W, Sepulveda RV, Gonzalez-Nilo F, Gonzalez C, Alvarez O (2017). Molecular determinants of BK channel functional diversity and functioning. Physiological Reviews.

[bib53] Lee S, Yu Y, Trimpert J, Benthani F, Mairhofer M, Richter-Pechanska P, Wyler E, Belenki D, Kaltenbrunner S, Pammer M, Kausche L, Firsching TC, Dietert K, Schotsaert M, Martínez-Romero C, Singh G, Kunz S, Niemeyer D, Ghanem R, Salzer HJF, Paar C, Mülleder M, Uccellini M, Michaelis EG, Khan A, Lau A, Schönlein M, Habringer A, Tomasits J, Adler JM, Kimeswenger S, Gruber AD, Hoetzenecker W, Steinkellner H, Purfürst B, Motz R, Di Pierro F, Lamprecht B, Osterrieder N, Landthaler M, Drosten C, García-Sastre A, Langer R, Ralser M, Eils R, Reimann M, Fan DNY, Schmitt CA (2021). Virus-induced senescence is a driver and therapeutic target in COVID-19. Nature.

[bib54] Li W, Aldrich RW (2004). Unique inner pore properties of BK channels revealed by quaternary ammonium block. The Journal of General Physiology.

[bib55] Li Q, Yan J (2016). Modulation of BK channel function by auxiliary beta and gamma subunits. International Review of Neurobiology.

[bib56] Li R-S, Xu G-H, Cao J, Liu B, Xie H-F, Ishii Y, Zhang C-F (2019). Alpha-mangostin ameliorates bleomycin-induced pulmonary fibrosis in mice partly through activating adenosine 5’-monophosphate-activated protein kinase. Frontiers in Pharmacology.

[bib57] Majdalawieh AF, Terro TM, Ahari SH, Abu-Yousef IA (2024). α-mangostin: a xanthone derivative in mangosteen with potent anti-cancer properties. Biomolecules.

[bib58] Martín P, Moncada M, Castillo K, Orsi F, Ducca G, Fernández-Fernández JM, González C, Milesi V (2021). Arachidonic acid effect on the allosteric gating mechanism of BK (Slo1) channels associated with the β1 subunit. Biochimica et Biophysica Acta. Biomembranes.

[bib59] McManus OB, Harris GH, Giangiacomo KM, Feigenbaum P, Reuben JP, Addy ME, Burka JF, Kaczorowski GJ, Garcia ML (1993). An activator of calcium-dependent potassium channels isolated from a medicinal herb. Biochemistry.

[bib60] McManus OB, Helms LM, Pallanck L, Ganetzky B, Swanson R, Leonard RJ (1995). Functional role of the beta subunit of high conductance calcium-activated potassium channels. Neuron.

[bib61] Morris GM, Huey R, Lindstrom W, Sanner MF, Belew RK, Goodsell DS, Olson AJ (2009). AutoDock4 and AutoDockTools4: Automated docking with selective receptor flexibility. Journal of Computational Chemistry.

[bib62] Nardi A, Olesen SP (2008). BK channel modulators: a comprehensive overview. Current Medicinal Chemistry.

[bib63] National Center for Biotechnology Information (2025). PubChem Compound Summary for CID 5281650, Mangostin. https://pubchem.ncbi.nlm.nih.gov/compound/Mangostin.

[bib64] Nordquist EB, Jia Z, Chen J (2024). Small molecule NS11021 promotes BK channel activation by increasing inner pore hydration. Journal of Chemical Information and Modeling.

[bib65] Orio P, Latorre R (2005). Differential effects of beta 1 and beta 2 subunits on BK channel activity. The Journal of General Physiology.

[bib66] Peiper U, Schmidt E (1972). Relaxation of coronary arteries by electro-mechanical decoupling or adrenergic stimulation. Pflugers Archiv European Journal of Physiology.

[bib67] Pereira da Silva EA, Martín-Aragón Baudel M, Navedo MF, Nieves-Cintrón M (2022). Ion channel molecular complexes in vascular smooth muscle. Frontiers in Physiology.

[bib68] Petiwala SM, Li G, Ramaiya A, Kumar A, Gill RK, Saksena S, Johnson JJ (2014). Pharmacokinetic characterization of mangosteen (Garcinia mangostana) fruit extract standardized to α-mangostin in C57BL/6 mice. Nutrition Research.

[bib69] Piechotta PL, Rapedius M, Stansfeld PJ, Bollepalli MK, Ehrlich G, Andres-Enguix I, Fritzenschaft H, Decher N, Sansom MSP, Tucker SJ, Baukrowitz T (2011). The pore structure and gating mechanism of K2P channels. The EMBO Journal.

[bib70] Piskorowski RA, Aldrich RW (2006). Relationship between pore occupancy and gating in BK potassium channels. The Journal of General Physiology.

[bib71] Ramaiya A, Li G, Petiwala SM, Johnson JJ (2012). Single dose oral pharmacokinetic profile of α-mangostin in mice. Current Drug Targets.

[bib72] Redhardt M, Raunser S, Raisch T (2024). Cryo-EM structure of the Slo1 potassium channel with the auxiliary γ1 subunit suggests a mechanism for depolarization-independent activation. FEBS Letters.

[bib73] Richter-Laskowska M, Trybek P, Delfino DV, Wawrzkiewicz-Jałowiecka A (2023). Flavonoids as modulators of potassium channels. International Journal of Molecular Sciences.

[bib74] Rockman ME, Vouga AG, Rothberg BS (2020). Molecular mechanism of BK channel activation by the smooth muscle relaxant NS11021. Journal of General Physiology.

[bib75] Roy S, Morayo Akande A, Large RJ, Webb TI, Camarasu C, Sergeant GP, McHale NG, Thornbury KD, Hollywood MA (2012). Structure-activity relationships of a novel group of large-conductance Ca(2+)-activated K(+) (BK) channel modulators: the GoSlo-SR family. ChemMedChem.

[bib76] Savineau JP, Marthan R (2000). Cytosolic calcium oscillations in smooth muscle cells. Physiology.

[bib77] Schewe M, Sun H, Mert Ü, Mackenzie A, Pike ACW, Schulz F, Constantin C, Vowinkel KS, Conrad LJ, Kiper AK, Gonzalez W, Musinszki M, Tegtmeier M, Pryde DC, Belabed H, Nazare M, de Groot BL, Decher N, Fakler B, Carpenter EP, Tucker SJ, Baukrowitz T (2019). A pharmacological master key mechanism that unlocks the selectivity filter gate in K^+^ channels. Science.

[bib78] Schrodinger LLC (2015). PyMOL.

[bib79] Shah KR, Guan X, Yan J (2021). Structural and functional coupling of calcium-activated BK channels and calcium-permeable channels within nanodomain signaling complexes. Frontiers in Physiology.

[bib80] Tao X, Hite RK, MacKinnon R (2017). Cryo-EM structure of the open high-conductance Ca2+-activated K+ channel. Nature.

[bib81] Tatiya-Aphiradee N, Chatuphonprasert W, Jarukamjorn K (2016). In vivo antibacterial activity of Garcinia mangostana pericarp extract against methicillin-resistant *Staphylococcus aureus* in a mouse superficial skin infection model. Pharmaceutical Biology.

[bib82] Tep-Areenan P, Suksamrarn S (2012). Mechanisms of vasorelaxation to gamma-mangostin in the rat aorta. Journal of the Medical Association of Thailand = Chotmaihet Thangphaet.

[bib83] Tykocki NR, Boerman EM, Jackson WF (2017). Smooth muscle ion channels and regulation of vascular tone in resistance arteries and arterioles. Comprehensive Physiology.

[bib84] Valverde MA, Rojas P, Amigo J, Cosmelli D, Orio P, Bahamonde MI, Mann GE, Vergara C, Latorre R (1999). Acute activation of Maxi-K channels (hSlo) by estradiol binding to the beta subunit. Science.

[bib85] Wang LW, Kang JJ, Chen IJ, Teng CM, Lin CN (2002). Antihypertensive and vasorelaxing activities of synthetic xanthone derivatives. Bioorganic & Medicinal Chemistry.

[bib86] Webb TI, Kshatri AS, Large RJ, Akande AM, Roy S, Sergeant GP, McHale NG, Thornbury KD, Hollywood MA (2015). Molecular mechanisms underlying the effect of the novel BK channel opener GoSlo: Involvement of the S4/S5 linker and the S6 segment. PNAS.

[bib87] Wu Y, Xiong Y, Wang S, Yi H, Li H, Pan N, Horrigan FT, Wu Y, Ding J (2009). Intersubunit coupling in the pore of BK channels. The Journal of Biological Chemistry.

[bib88] Wu RS, Marx SO (2010). The BK potassium channel in the vascular smooth muscle and kidney: α- and β-subunits. Kidney International.

[bib89] Xin J, Jiang X, Ben S, Yuan Q, Su L, Zhang Z, Christiani DC, Du M, Wang M (2022). Association between circulating vitamin E and ten common cancers: evidence from large-scale Mendelian randomization analysis and a longitudinal cohort study. BMC Medicine.

[bib90] Xu Y, Wu J, Gao L, Lin H, Yang Z, Liu X, Niu Y (2024). α-Mangostin reduces hypertension in spontaneously hypertensive rats and inhibits EMT and fibrosis in Ang II-induced HK-2 cells. International Journal of Medical Sciences.

[bib91] Yamanouchi D, Kasuya G, Nakajo K, Kise Y, Nureki O (2023). Dual allosteric modulation of voltage and calcium sensitivities of the Slo1-LRRC channel complex. Molecular Cell.

[bib92] Zhang G, Xu X, Jia Z, Geng Y, Liang H, Shi J, Marras M, Abella C, Magleby KL, Silva JR, Chen J, Zou X, Cui J (2022). An allosteric modulator activates BK channels by perturbing coupling between Ca2+ binding and pore opening. Nature Communications.

[bib93] Zhou Y, Xia XM, Lingle CJ (2011). Cysteine scanning and modification reveal major differences between BK channels and Kv channels in the inner pore region. PNAS.

[bib94] Zhuge R, Fogarty KE, Tuft RA, Walsh JV (2002). Spontaneous transient outward currents arise from microdomains where BK channels are exposed to a mean Ca(2+) concentration on the order of 10 microM during a Ca(2+) spark. The Journal of General Physiology.

